# Aripiprazole as a Candidate Treatment of COVID-19 Identified Through Genomic Analysis

**DOI:** 10.3389/fphar.2021.646701

**Published:** 2021-03-02

**Authors:** Benedicto Crespo-Facorro, Miguel Ruiz-Veguilla, Javier Vázquez-Bourgon, Ana C. Sánchez-Hidalgo, Nathalia Garrido-Torres, Jose M. Cisneros, Carlos Prieto, Jesus Sainz

**Affiliations:** ^1^ Department of Psychiatry, School of Medicine, University Hospital Virgen del Rocio-IBIS, Sevilla, Spain; ^2^ Spanish Network for Research in Mental Health (CIBERSAM), Sevilla, Spain; ^3^ Department of Psychiatry, University Hospital Marques de Valdecilla - Instituto de Investigacion Marques de Valdecilla (IDIVAL), Santander, Spain; ^4^ Department of Medicine and Psychiatry, School of Medicine, University of Cantabria, Santander, Spain; ^5^ Seville Biomedical Research Centre (IBiS), Sevilla, Spain; ^6^ Department of Infectious Diseases, Microbiology and Preventive Medicine, Institute of Biomedicine of Seville, University Hospital Virgen del Rocio, University of Seville, Salamanca, Spain; ^7^ Spanish Network for Research in Infectious Diseases (REIPI), Madrid, Spain; ^8^ Bioinformatics Service, Nucleus, University of Salamanca, Salamanca, Spain; ^9^ Spanish National Research Council (CSIC), Institute of Biomedicine and Biotechnology of Cantabria, Santander, Spain

**Keywords:** psychosis, inflammation, immunology, coronavirus, repurposing drugs, elopiprazole, SARS-CoV-2

## Abstract

**Background:** Antipsychotics modulate expression of inflammatory cytokines and inducible inflammatory enzymes. Elopiprazole (a phenylpiperazine antipsychotic drug in phase 1) has been characterized as a therapeutic drug to treat SARS-CoV-2 infection in a repurposing study. We aim to investigate the potential effects of aripiprazole (an FDA approved phenylpiperazine) on COVID-19-related immunological parameters.

**Methods:** Differential gene expression profiles of non-COVID-19 vs. COVID-19 RNA-Seq samples (CRA002390 project in GSA database) and drug-naïve patients with non-affective psychosis at baseline and after three months of aripiprazole treatment were identified. An integrative transcriptomic analyses of aripiprazole effects on differentially expressed genes in COVID-19 patients was performed.

**Findings:** 82 out the 377 genes (21.7%) with expression significantly altered by aripiprazole have also their expression altered in COVID-19 patients and in 93.9% of these genes their expression is reverted by aripiprazole. The number of common genes with expression altered in both analyses is significantly higher than expected (Fisher’s Exact Test, two tail; *p* value = 3.2e-11). 11 KEGG pathways were significantly enriched with genes with altered expression both in COVID-19 patients and aripiprazole medicated non-affective psychosis patients (*p* adj<0.05). The most significant pathways were associated to immune responses and mechanisms of hyperinflammation-driven pathology (i.e.,“inflammatory bowel disease (IBD)” (the most significant pathway with a *p* adj of 0.00021), “Th1 and Th2 cell differentiation” and “B cell receptor signaling pathway”) that have been also associated with COVID19 clinical outcome.

**Interpretation:** This exploratory investigation may provide further support to the notion that a protective effect is exerted by aripiprazole (phenylpiperazine) by modulating the expression of genes that have shown to be altered in COVID-19 patients. Along with many ongoing studies and clinical trials, repurposing available medications could be of use in countering SARS-CoV-2 infection, but require further studies and trials.

## Introduction

The SARS-CoV-2 epidemic has become the greatest challenge facing medicine today. Infected patients present with a wide range of clinical severity varying from asymptomatic to fatal condition (Wu et al., 2020). Advanced age, gender (male) and suffering comorbidities (diabetes, cardiovascular or chronic respiratory diseases) are risk factors for higher clinical severity, hospitalization rate and death from COVID-19 (Rubino et al., 2020; Zhou et al., 2020). The presence of these comorbidities may decrease resilience and lower the ability to tolerate additional cytokine storm (Mangalmurti and Hunter, 2020).

COVID-19 individuals who become critically and fatally ill seem to experience an indiscriminate and runaway immune response with an unchecked systemic overproduction of cytokines and immunological disbalance (Bhaskar et al., 2020; Manjili et al., 2020; Zeng et al., 2020). COVID-19 related immunopathogenesis is not understood just as an emergent cytokine storm but also as an impairment of protective T cell immunity (Chen et al., 2020).

There are no FDA-approved antivirals or vaccines for any coronavirus, including SARS-CoV-2, and current treatments for COVID-19 are limited to supportive therapies and off-label use of FDA-approved drugs. Anti-inflammatory drugs such as dexamethasone have been shown to reduce deaths (Vabret et al., 2020). The crucial role of NLRP3 inflammasome activation in the pathogenesis of diseases caused by SARS-CoVs draws also attention toward potential role of its inhibitors in the treatment of COVID-19 (van den Berg and Te Velde, 2020). Wide range of different drug classes, such as cancer therapeutics, antipsychotics, and antimalarials, seem to have a beneficial effect against MERS and SARS coronaviruses (Dyall et al., 2017). Weston et al., (2020) observed that, although infection cannot be prevented, chlorpromazine (typical antipsychotic drug) and chloroquine protect mice from severe clinical disease from SARS-CoV. Clozapine (atypical antipsychotic drug) has revealed to be effective in suppressing the proinflammatory cytokine expression by limiting the NLRP3 inflammasome activation *in vitro* (Giridharan et al., 2020). In the same line as above, Riva and colleagues (2020) analyzed approximately 12,000 drugs in clinical-stage or Food and Drug Administration (FDA)-approved small molecules to identify candidate drugs to treat COVID-19 and reported that elopiprazole (a never marketed phenylpiperazine antipsychotic drug) was listed among the 21 most potent compounds to inhibit SARS-CoV infection. Phenyl-piperazine derivatives had proved their utility as an effective source of antiviral compounds, with different mechanisms of action, for treatment of human adenovirus and cytomegalovirus (DNA viruses) (Sanchez-Cespedes et al., 2016).

Antipsychotics suppress expression of inflammatory cytokines and inducible inflammatory enzymes (i.e., cyclooxygenase) and microglia activation (Dinesh et al., 2020). These anti-inflammatory effects are elicited via the reduction of proinflammatory cytokines production, modulating monocytes response through TLR and the inhibition of the microglial activation by reducing the levels of inducible nitric oxide synthase (iNOS), IL-1β, IL-6, and TNF-α (Kato et al., 2007; Obuchowicz et al., 2017). In humans, the immunomodulatory effect of risperidone (pyridopyrimidines) and aripiprazole (marketed phenylpiperazine) has been demonstrated (Juncal-Ruiz et al., 2018), with aripiprazole demonstrating a greater anti-inflammatory effect on TNF-α, IL-13, IL-17α and fractalkine. Thus, the protective effect of phenylpiperazine marketed antipsychotics (aripiprazole) against a pernicious cytokine storm is a hypothesis that warrants further investigation with the aim of unrevealing new off-label drug to be use in severe COVID19 patients.

Prevalence and severity of COVID-19 infection in patients with severe mental disorders have yielded to inconsistent results, likely due to differences in the methodology utilized in these investigations. Lee and collaborators (2020) reported that diagnosis of a mental disorder was not associated with increased likelihood of SARS-CoV infection, but a slightly higher risk for severe clinical outcomes (Lee et al., 2020). Wang and colleagues (2021) reported a higher overall risk to get infected among schizophrenia patients. In a retrospective epidemiological study, we observed that vulnerable severe mental disorder individuals on long-acting injectable antipsychotics had a lower risk of SARS-CoV2 infection and a better outcome after infection (unpublished data).

The aim of the present study was to examine the potential beneficial effects of aripiprazole (antipsychotic) in COVID-19 infection by: 1.- analyzing the profile of gene expression of drug-naïve patients with psychosis at baseline and after three months of treatment with aripiprazole (PAFIP sample); and 2.- comparing the set of genes with altered expression in COVID-19 patients (Wuhan sample) with the set of genes modulated by aripiprazole in drug-naïve schizophrenia patients.

## Materials and Methods

### PAFIP Cohort of Aripiprazole-Treated Patients

#### Setting and Sample Study

The cohort analyzed to study the effect of aripiprazole was obtained at the University Hospital Marques de Valdecilla (Cantabria, Spain). Conforming to international standards for research ethics, this study was approved by the Cantabria Ethics Institutional Review Board (IRB). Patients meeting inclusion criteria for a first episode of non-affective psychosis (drug-naïve) provided written informed consent to be included in the study. After informed consent was signed, patients were included in a prospective, randomized, flexible-dose, open-label study (Crespo-Facorro et al., 2017; Mayoral Van-Son et al., 2021).

#### Laboratory Assessments

Blood samples were obtained from 57 fasting non-affective psychosis subjects (25 males and 32 females; mean age of 31.54 years) from 8:00 to 10:00 a.m. by the same staff and in the same setting. A detailed description of methodology followed to assess biochemical variables is available upon request to the authors. None of the patients had a chronic inflammation or infection, or were taking medication that could influence the results of blood tests.

#### RNA Extraction

Total RNA was extracted from blood using the Tempus™ Blood RNA Tube and the Tempus™ Spin RNA Isolation Kit (Applied Biosystems, Foster City, CA, United States) following the manufacturer’s protocols. To select only high-quality RNA, the RNA integrity number (RIN) was characterized with a Bioanalyzer (Agilent Technologies, Santa Clara, CA, United States) and samples with a RIN of at least 7.6 were used.

#### RNA Next-Generation Sequencing

Total RNA was extracted from peripheral blood of each individual. The messenger RNA (mRNA) obtained from blood was sequenced at the Centro Nacional de Análisis Genómico (CNAG) using Illumina HiSeq instruments (San Diego, CA, United States). The mRNA was isolated from the total RNA and was fragmented once transformed into complementary DNA (cDNA). Fragments of 300bp on average were selected to construct the cDNA libraries for sequencing. Pair-end sequences of 70 nucleotides for each end were produced. The mRNA from blood samples of 57 drug-naïve non-affective psychosis patients at baseline and after 3 months of continuous treatment with aripiprazole was sequenced.

Sequence files were aligned to the GRCh38 human reference genome (Gencode release 25) using the STAR aligner (Harrow et al., 2012; Dobin et al., 2013). Reads count were normalized with the Voom algorithm using the cyclic loess method (Law et al., 2014) and significant gene expression changes between treated and naïve patients were identified with lima (Smyth et al., 2005). We performed a paired analysis using a Wald test and a parametric fit type with an adjusted *p*-value cutoff of 0.01.

### Wuhan COVID19 Dataset

COVID data was downloaded from the GSA server (Wang et al., 2017) with the CRA002390 identifier (Xiong et al., 2020). This research collected RNA-Seq samples of peripheral blood mononuclear cells (PBMC) from three COVID-19 patients (all were males; mean age of 45.3 years) and three healthy donors at Zhongnan Hospital of Wuhan University that were included in the present study. These data were analyzed with RaNA-Seq (Prieto and Barrios, 2020) cloud platform and differential expression genes were detected by means of DESeq2 (Love et al., 2014) cloud using a Wald test, a parametric fit type and setting an adjusted *p*-value cutoff of 0.01.

### Statistical and Bioinformatic Analysis

Statistical significance of differential expression co-occurrence between COVID-19 and aripiprazole studies was calculated with a Fisher Exact test. Functional enrichment analyses, to identify biological pathways in the Biosystem database (downloaded on January 2020) (Geer et al., 2010) with a significant presence of differential expressed genes, were carried out with a Fisher Exact test. KEGG was used for representation and analysis of molecular networks (Kanehisa et al., 2010). Visualization and final representation of pathways were performed with the pathview R package (Luo and Brouwer, 2013).

## Results

Integrative transcriptomic analyses of aripiprazole effects on differentially expressed genes in COVID-19 patients:

### COVID-19 Patients Versus Healthy Donors (Wuhan Sample)

We found 2,137 genes with significant differential expression between COVID-19 patients and controls (*p* adj value < 0.05) (Supplementary Table S1). The most significant gene was the Charcot-Leyden crystal galectin gene (CLC) with a *p* adj value of 7.8e-23.

### Drug-Naïve Non-affective Psychosis Patients at Entry and After 3 months of Aripiprazole Treatment (PAFIP Sample)

We found 377 genes with significant differential expression before and after medication (*p* adj value < 0.05) (Supplementary Table S2). The two most significant genes were the LIM domain only 4 (LMO4) and the ATP binding cassette subfamily A member 9 (ABCA9) with a *p* adj value of 0.0039.

### Differential Gene Expression Between COVID-19 and Aripiprazole-Treated Samples

**FIGURE 1:**
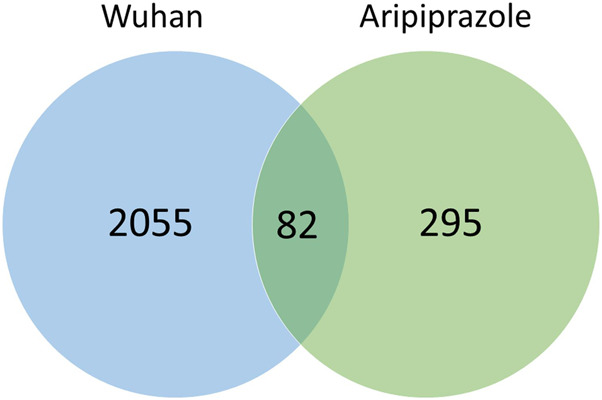
Number of genes with expression altered in Wuhan sample (COVID-19) and PAFIP sample (aripiprazole-treated).

82 out the 377 genes (21.7%) modified by aripiprazole treatment (PAFIP sample) are significantly also altered in COVID-19 patients (Wuhan sample) (Figure 1). The number of common genes to both analyses is significantly higher than expected by chance (Fisher’s Exact Test, two tail; *p* value = 3.2e-11).

Interestingly, out of the 82 genes with expression altered in both analyzed cohorts 55 genes have decreased expression after aripiprazole medication and increased expression in COVID-19 patients; also, out of the 82 genes common to both cohorts 22 have increased expression after aripiprazole medication and decreased expression in COVID-19 patients. In total 77 genes out of 82 (93.9%) have altered expression in different direction when we compared the effects of COVID-19 and aripiprazole medication.

### KEGG Pathways Significantly Enriched With Common Genes With Altered Expression in COVID-19 and Aripiprazole-Treated Samples

The analysis of pathways for enrichment of common genes with altered expression in COVID-19 and aripiprazole patients shows 11 pathways significantly enriched (*p* value Fisher <0.05) (Table 1). Several of those pathways are related to the immune system such as the “inflammatory bowel disease (IBD)” (the most significant pathway; *p* adj of 0.00021), “Th1 and Th2 cell differentiation” and “B cell receptor signaling pathway,” both related to the defense against infections.

**TABLE 1:** KEGG pathways significantly enriched with genes with altered expression in COVID19 patients and schizophrenia patients treated with aripiprazole.

Pathway ID	Source	Pathway name	Observed %	Expected %	*p* value Fisher	No. of genes per pathway	Gene symbol
hsa05321	KEGG	Inflammatory bowel disease (IBD)	8.16	0.57	0.00021	4	*GATA3 HLA-DQB1 IL18 STAT4*
hsa04658	KEGG	Th1 and Th2 cell differentiation	8.16	0.82	0.00079	4	*GATA3 HLA-DQB1 NFATC2 STAT4*
hsa04380	KEGG	Osteoclast differentiation	8.16	1.17	0.00281	4	*BTK NFATC2 SYK LILRB4*
hsa04664	KEGG	Fc epsilon RI signaling pathway	6.12	0.62	0.00382	3	*BTK PLA2G4A SYK*
hsa04662	KEGG	B cell receptor signaling pathway	6.12	0.65	0.00430	3	*BTK NFATC2 SYK*
hsa05164	KEGG	Influenza A	8.16	1.50	0.00653	4	*HLA-DQB1 HLA-DQB1 IL18 NLRP3*
hsa04064	KEGG	NF-kappa B signaling pathway	6.12	0.84	0.00867	3	*BTK LTA SYK*
hsa04659	KEGG	Th17 cell differentiation	6.12	0.95	0.01202	3	*GATA3 HLA-DQB1 NFATC2*
hsa04611	KEGG	Platelet activation	6.12	1.09	0.01712	3	*BTK PLA2G4A SYK*
hsa05166	KEGG	HTLV-I infection	8.16	2.33	0.02787	4	*FZD2 HLA-DQB1 LTA NFATC2*
hsa05152	KEGG	Tuberculosis	6.12	1.53	0.03986	3	*HLA-DQB1 IL18 SYK*

## Discussion

Our results herein revealed that the differently expressed genes in COVID-19 patients and schizophrenia patients treated with aripiprazole were highly associated with numerous immune-related pathways, including “Inflammatory bowel disease (IBD)”, “Th1 and Th2 cell differentiation”, “Fc epsilon RI signaling pathway”, “B cell receptor signaling pathway”, “NF-kappa B signaling pathway” and “Th17 cell differentiation”. It is worth to remark that these immunological pathways have been associated with COVID19 clinical outcome. Thus, a wide array of host humoral and cellular immune response alterations associated with SARS-CoV-2 infection might cause an uncontrolled or insufficient immune response that may lead to immunopathology and cause severe damage to patients. (Tay et al., 2020; Wang et al., 2021). Lymphopenia marked by T cell and NK cell dysfunction, increases in proinflammatory markers and cytokines, and potential blood hypercoagulability characterize severe COVID-19 cases (Vabret et al., 2020).

In more severe COVID-19 cases, death results from hypoxemic respiratory failure in patients developing severe acute respiratory distress syndrome and is associated, in a substantial portion of patients, with an inflammatory syndrome and cytokine storm (Mehta et al., 2020) that may originate from immune cells (Chen et al., 2020). Longitudinal analysis of the immune response observed in a fatal case of COVID-19 revealed waves of a pro-inflammatory cytokine storm, Th1 and Th2 activation, and markers of T cell exhaustion, apoptosis, cell cytotoxicity, and endothelial activation were until the fatal outcome (Bouadma et al., 2020).

The TH17 type response profoundly also contributes to the cytokine storm in pulmonary viral infection including SARS-CoV-2 (Josset et al., 2013). Compared with non-ICU COVID-19 patients, ICU COVID-19 patients have even higher levels of several cytokines specifically involved in TH17 type responses (Huang C. et al., 2020). It has been recently proposed that JAK2 inhibitor (Fedratinib@) can prevent the deteriorating outcomes of TH17 associated cytokine storm in COVID-19 by suppressing the production of several TH17 signature cytokines (Wu and Yang, 2020). Targeting the TH17 pathway may benefit the patients with TH17 dominant immune profiles.

Among biologic agents in patients with severe COVID-19 inhibiting Fc epsilon RI signaling has been proposed (Yalcin and Yalcin, 2021). Omalizumab specifically binds to the CH3 domain, is near to the binding site for the high affinity IgE Fc receptors type-I (also called FceRI) of human IgE (Metz et al., 2017). A critical issue in patients with Covid-19 is the viremia and the overresponse to this viremia with increase of ferritin, CRP and D-Dimer that are directly associated with the mortality (Huang I. et al., 2020). It has been described that omalizumab safely decreases the coagulant proteins (D-Dimer) and proinflammatory cytokines/mediators and increases the anti-coagulant proteins (protein C, S) in patients with sepsis (Yalcin et al., 2013; Criado et al., 2020). So, it could be anticipated that we may administer it for severe COVID-19 (Yalcin and Yalcin, 2021).

We observed that those genes differentially expressed in COVID-19 and aripiprazole-treated patients were involved in Inflammatory bowel disease (IBD). Autoinflammatory diseases (IBD) that present with bowel inflammation and intractable diarrhea owing to an inappropriate inflammatory response, with also altered key immune pathways underlying persistent inflammation such as excessive IL-1 signaling, constitutive NF-κB activation, and chronic type I IFN signaling (de Jesus et al., 2015). It is also of interest that JAK-STAT pathways, as well as in COVID19, play an important role in the inflammatory response characteristic of IBD and represent a promising therapeutic target for treatment of the disease. (Pedersen et al., 2014).

In our study, differentially expressed genes involved in NF-kappa B signaling pathway were identified according to KEGG pathway analysis. The activated NF-κB transcription factors serve as a “rapid acting” primary transcription factor regulating diverse cellular responses associated with chronic inflammatory states, septic shock syndrome and multiorgan failure (Zhang et al., 2017). Hyper-activation of the nuclear factor kappa-light-chain enhancer of activated B cells (NF-κB) pathway has been implicated in the pathogenesis of the severe/critical COVID19 phenotype (Hirano and Murakami, 2020). Cromolyn, which inhibit NF-κB mediated cytokine production, has been suggested as a repurposing drug in the fight against COVID-19 (Karadsheh Adli, 2020). Many of the drugs currently effective in COVID disease appear to have links to the NF-κB cascade of immune regulation (Hariharan et al., 2020).

Despite all recent generated scientific knowledge, immune responses and mechanisms of hyperinflammation-driven pathology need to be further elucidated to address how these immune differences across patients or between different types of coronavirus infections dictate who succumbs to disease and who remains asymptomatic (Vabret et al., 2020).

**FIGURE 2:**
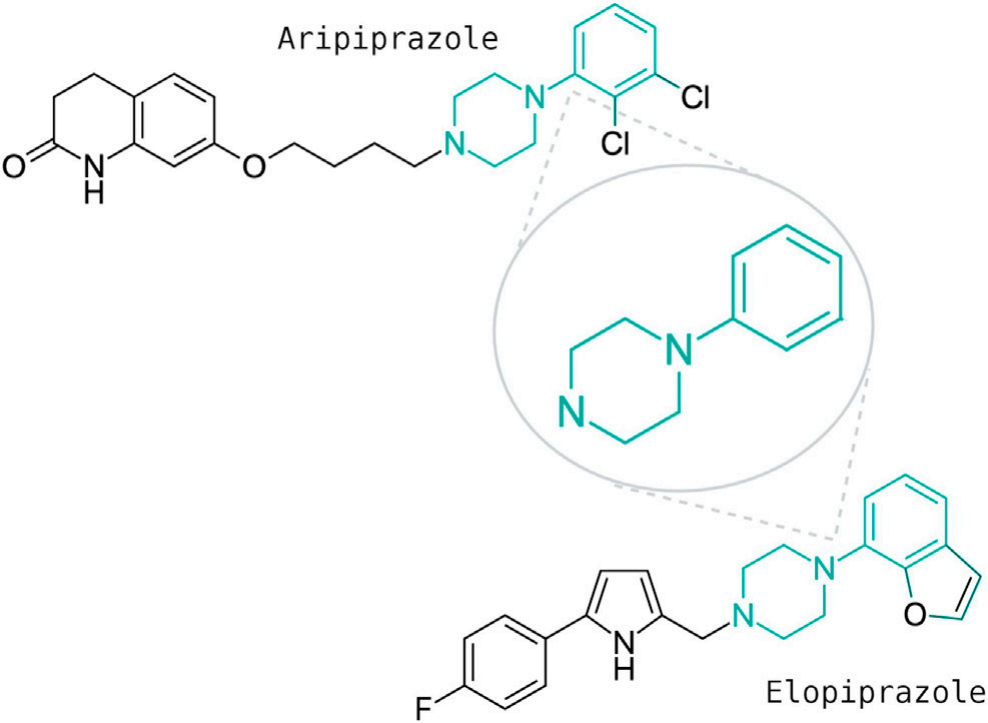
Chemical structures of two phenypiperazines with antipsychotic effects: elopiprazole and aripiprazole.

The fact that antipsychotics have a demonstrated effect on immunological pathways, inducible inflammatory enzymes (i.e., cyclooxygenase), and microglia activation (Dinesh et al., 2020) may lead to the speculation about the beneficial effects of these drugs on controlling the acute hyper-inflammatory response that may be responsible for critical COVID19 illness. A recent study (Riva et al., 2020) profiled a library of approximately 12,000 drugs in clinical-stage or FDA-approved small molecules to identify candidate drugs to treat COVID-19. In the list of the 21 most potent compounds to inhibit infection validated in dose response across multiple cell lines there are two antipsychotic drugs, elopiprazole and 8-(3-Chlorostyryl) caffeine which are in phase I and preclinical stages respectively. Elopiprazole and aripiprazole belong to the class of organic compounds known as phenylpiperazines, containing a phenylpiperazine skeleton, which consists of a piperazine bound to a phenyl group (Figure 2).

If our results herein prove to be certain one may expect a milder severity of COVID-19 in patients on aripiprazole (phenylpiperazine). Differences in the definitions and methodology utilized in recent investigations have yielded to inconsistent results about the prevalence and severity of COVID-19 infection among patients with severe mental disorders (Lee et al., 2020; Wang et al., 2021). It is of worth to pinpoint that Wang and colleagues (2021) reported that chronic schizophrenia patients have a lower association compared with recent diagnosis of schizophrenia (less than 1 year) (AOR = 1.48, 95% CI: 1.33–1.65 vs. AOR = 9.89, 95% CI: 8.68–11.26), giving place to the speculation that antipsychotic treatment may exert a protective effect. Nemani et al., (2021) recently described that schizophrenia spectrum diagnosis was associated with an increase of death or discharge to hospice outcome at 45 days following testing and highlighted the relevance of evaluating the potential protective effects of psychotropic medications. Our data retrospectively exploring an epidemiological sample of people with severe mental disorders who are on long-acting injectable antipsychotics revealed a lower risk of SARS-CoV2 infection and a better outcome after infection in this group of antipsychotic treated individuals compared to overall COVID-19 people (Canal-Rivero et al., 2021).

Some limitations need to be taken into when interpreting our results. First, differences in gender distribution between cohorts may limit the comparability of gene expression data. Second, the pattern of gene expression associated to COVID19 infection raises from three COVID19 patients and three healthy donors from whom there is limited access to subjects’ characteristics.

## Conclusion

Given that the effect of aripiprazole in gene expression mainly revert the changes in expression caused by COVID19 infection, and that another never marked phenylpiperazine antipsychotic (elopiprazole) has been validated as potential treatment for COVID19, it may be suggested that aripiprazole might be used as treatment for COVID19. Along with many ongoing studies and clinical trials, repurposing available medications could be of use in countering SARS-CoV-2 infection, but clearly require further studies and trials.

## Data Availability

The datasets presented in this study can be found in online repositories. The names of the repository/repositories and accession number(s) can be found below: https://www.ebi.ac.uk/ena, PRJEB42627.
