# Supplementary material for: Aripiprazole as a Candidate Treatment of COVID-19 Identified Through Genomic Analysis
Source: Front Pharmacol. 2021 Mar 2;12:646701. doi: 10.3389/fphar.2021.646701 (PMC7982825; doi:10.3389/fphar.2021.646701)
Supplement: Supplementary file 1 [file datasheet1.pdf]

Table S1. Differential expression between COVID19 patients and controls

Gene Symbol	Base Mean Control	Base Mean COVID-19 patients	P adj	Gene Symbol	Base Mean Control	Base Mean COVID-19 patients	P adj
CLC	67.869	0.915	7.8e-23	AL139274.1	1.784	0.15	0.008
PHRF1	0	7.788	9.4e-21	FAM105A	22.103	37.542	0.008
ADAMTS2	0.423	67.785	1.6e-20	CTSL	6.65	16.611	0.008
HLA-A	1.725	818.39	1.4e-19	ASL	14.48	18.009	0.008
C2	0.213	1.92	8.6e-19	RHEB	25.075	36.029	0.009
MAML1	12.378	6.514	1.5e-18	SBSPON	2.487	0.789	0.009
RPS9	11.475	169.524	1.6e-18	MTHFD2	14.603	20.313	0.009
CROCC2	2.687	0.049	3.0e-18	EDNRB	0.016	0.457	0.009
IGHV1-2	1.579	117.506	1.0e-17	GALNT2	16.885	29.859	0.009
IKZF2	15.732	3.234	1.0e-17	CENPA	0.627	1.254	0.009
TPX2	0.594	2.767	2.3e-16	COPA	54.259	69.981	0.009
RASSF7	1.42	4.541	8.6e-16	TRBV29-1	102.253	17.957	0.009
RPP21	0.055	8.764	2.9e-14	MRPS7	16.492	22.795	0.009
IGLV7-46	2.523	53.729	4.1e-13	SLC9A7P1	1.1	3.131	0.009
HCG4P5	0	5.889	5.0e-13	CHRNE	3.069	0.765	0.009
CDKN1C	7.194	0.494	5.2e-13	TPM2	20.948	5.583	0.009
CD180	0.837	2.04	1.2e-12	MTND1P23	98.483	26.206	0.009
HCG4P5	0.029	26.521	2.8e-12	UBTD1	3.716	6.566	0.009
SLC25A29	56.283	17.038	3.0e-12	KCNJ3	0.576	0.051	0.009
MYBL2	0.862	8.552	3.3e-12	RAB39A	1.509	2.592	0.009
DDX39B	0	2.504	3.3e-12	TMEM181	25.547	9.794	0.009
B3GALT4	3.077	0	3.4e-12	GLRX	64.442	136.617	0.009
CHST13	1.027	5.25	5.6e-12	TPPP	1.316	0.234	0.009
FN1	2.547	17.781	5.8e-12	DNASE1L1	16.687	24.676	0.009
DLGAP5	0.248	1.618	1.0e-11	SLC35A1	14.871	21.685	0.009
CES1	0.567	30.254	2.9e-11	IGHJ2	1.447	107.905	0.009
SIGLEC17P	12.732	1.28	9.7e-11	AC073850.1	2.424	16.142	0.009
CDC25A	0.069	0.837	1.0e-10	RNF135	24.005	33.048	0.009
AC008695.1	0	0.494	1.3e-10	SFTPD	0.904	0.098	0.009
IGHG1	12.891	2226.887	1.8e-10	DBI	99.497	136.81	0.009
IL5RA	4.983	0.229	3.5e-10	TIMM23	11.405	14.657	0.009
C2	1.678	8.219	3.9e-10	MELK	0.486	1.617	0.009
KIF20A	0.415	1.284	3.9e-10	AURKB	3.83	6.467	0.009
LIPA	34.561	72.45	4.2e-10	TMEM60	4.789	6.382	0.009
YPEL1	16.44	5.427	4.9e-10	PRAF2	15.961	6.731	0.009
BUB1	0.807	2.96	8.3e-10	TMEM51	0.171	0.527	0.009
CCR3	12.779	0.409	8.4e-10	ZNF540	2.561	1.156	0.009
CPA3	7.28	0.177	9.4e-10	UQCR10	42.322	61.687	0.009
CDCA5	0.531	3.258	1.1e-9	DMRTC2	0.54	0.054	0.009
IGKV3-20	44.809	572.206	1.2e-9	ITGA1	1.512	2.323	0.009
CDC20	0.994	5.002	1.4e-9	NAGA	21.77	44.485	0.009
ASPM	0.175	0.969	1.4e-9	WDR45P1	0.15	1.016	0.009
IGHV4-39	14.089	556.548	1.9e-9	HLA-H	0.293	0	0.009
TOP2A	1.208	3.166	2.0e-9	STAG3L2	18.635	8.468	0.009
C1QC	0.356	8.089	5.5e-9	SPR	0.771	1.754	0.009
IGKV3D-20	1.45	24.568	5.5e-9	MARCKSL1	28.277	8.906	0.009
MAOA	0.044	14.276	6.3e-9	DBNDD2	7.95	14.694	0.009
KIF4A	0.072	0.578	6.4e-9	ETFA	37.798	50.108	0.009
VSIG4	1.406	77.693	6.5e-9	MSH3	3.59	5.187	0.009

Table S1. Continuation

Gene Symbol	Base Mean Control	Base Mean COVID-19 patients	P adj	Gene Symbol	Base Mean Control	Base Mean COVID-19 patients	P adj
SIGLEC16	1.205	7.221	6.9e-9	IFNGR2	22.158	52.356	0.009
CCL4L2	15.391	0.922	7.0e-9	EBF4	4.853	0.994	0.009
AGAP1	8.632	1.306	7.0e-9	TBCC	29.178	12.48	0.009
ZNF266	56.064	14.047	7.0e-9	TRPM2	7.82	16.992	0.009
CCNB2	0.784	3.796	7.5e-9	POLDIP2	20.772	28.116	0.009
FAM20C	1.862	11.872	7.7e-9	ARID4A	43.743	22.15	0.009
MGST1	8.711	62.114	8.3e-9	DRAM2	63.826	88.331	0.009
CBR1	7.323	14.608	9.6e-9	UPP1	57.739	97.931	0.009
SIGLEC11	0.617	3.384	1.0e-8	AP000936.3	15.753	2.086	0.009
ALDH4A1	1.805	4.204	1.1e-8	AL079341.1	0.792	0.053	0.009
RNASE1	0.609	44.436	1.1e-8	AL365205.1	1.259	2.707	0.009
CRY1	14.088	4.131	1.2e-8	AC125612.1	4.174	0.22	0.01
BIRC5	0.592	3.506	1.5e-8	ADAM9	9.595	22.247	0.01
PDZD4	40.726	7.012	1.5e-8	GPR68	7.432	2.29	0.01
CENPW	1.14	5.281	1.5e-8	GAA	67.41	129.297	0.01
ME1	0.294	2.004	1.7e-8	ATP5PD	107.554	147.894	0.01
IGHV3-20	1.605	76.446	1.8e-8	MSC	3.362	0.651	0.01
CDHR1	1.575	0.41	1.8e-8	JAK2	19.612	36.43	0.01
CES1	4.525	118.134	2.3e-8	KNDC1	1.023	0.124	0.01
NUAK1	3.114	0.192	2.6e-8	PPFIA3	1.983	0.768	0.01
HJURP	1.286	2.656	3.6e-8	CYB5R3	34.646	44.472	0.01
SLC1A3	0.444	11.222	4.5e-8	MAN2B1	117.035	179.289	0.01
MRC1	0.14	3.574	4.6e-8	RPN1	64.561	80.092	0.01
KIR2DP1	1.462	0	5.0e-8	ERMAP	8.703	13.955	0.01
MKI67	0.975	4.52	5.0e-8	ZNF354B	42.186	8.017	0.01
NUSAP1	3.379	9.746	5.7e-8	PSEN2	4.496	6.379	0.01
MYBL1	50.918	7.218	7.3e-8	S100A10	306.426	449.828	0.01
PTGFRN	0.243	1.062	7.6e-8	GLB1	39.298	56.111	0.01
GLI3	0.297	0.033	7.6e-8	HAUS1P1	0.296	1.059	0.01
MPDU1	23.066	43.619	7.7e-8	AC007182.2	1.138	6.808	0.01
IGKV2-30	13.39	131.965	8.7e-8	IGLVI-70	0.615	3.9	0.01
TK1	1.93	5.606	8.7e-8	HEBP1	16.619	24.928	0.01
IGHA1	111.174	450.344	1.2e-7	BAX	87.087	108.045	0.01
ACCS	19.879	66.17	1.2e-7	VDAC2	66.372	69.505	0.01
SHCBP1	1.756	5.631	1.2e-7	BCL11B	36.814	7.36	0.01
C16orf45	5.486	1.523	1.3e-7	CD200R1	2.155	0.531	0.01
ACVRL1	0.664	2.526	1.3e-7	AC005912.1	6.229	0.573	0.01
RXRB	6.649	0.012	1.3e-7	KLRD1	55.809	26.128	0.01
NT5DC2	2.99	17.851	1.4e-7	STAT4	83.239	23.27	0.01
MMP23B	21.532	6.637	1.5e-7	PAQR4	4.157	7.152	0.01
FCAR	4.868	9.246	1.7e-7	BRK1	80.32	105.702	0.01
IGKV1-16	5.281	68.039	1.7e-7	CFH	4.608	0.935	0.01
KIF11	0.739	1.785	1.7e-7	CHI3L1	37.645	3.577	0.01
ATP5F1B	226.415	293.567	1.9e-7	TRAF7	25.032	35.54	0.011
MS4A2	8.159	0.903	2.3e-7	NDUFB10	58.135	78.072	0.011
CACNG6	1.665	0.044	2.4e-7	TTC39B	19.273	8.659	0.011
BTG1P1	20.072	2.207	3.1e-7	PGD	111.061	252.797	0.011
MRPL37	18.152	30.117	4.1e-7	P3H4	5.731	2.663	0.011
AC256203.2	2.121	0.41	4.1e-7	WNT11	0.261	1.331	0.011
B3GALT4	2.381	4.978	4.1e-7	GATB	4.983	7.655	0.011
TRDC	115.054	24.886	4.1e-7	ZBTB32	1.01	3.127	0.011

Table S1. Continuation

Gene Symbol	Base Mean Control	Base Mean COVID-19 patients	P adj	Gene Symbol	Base Mean Control	Base Mean COVID-19 patients	P adj
SLCO4A1	0.72	2.049	4.1e-7	TTC38	48.364	23.564	0.011
TSPYL1	55.526	17.472	4.2e-7	ADAMDEC1	0.102	0.482	0.011
IGKV1D-39	26.08	803.531	4.2e-7	ANK3	6.55	1.912	0.011
IGKV2-28	16.808	629.318	4.6e-7	CD226	49.165	24.979	0.011
HLA-K	0	1.435	5.3e-7	MARCO	5.924	18.361	0.011
RNASE2	10.487	191.492	5.3e-7	APOBEC3A	0.092	1.076	0.011
LTA	0	1.245	5.6e-7	PRF1	152.883	47.449	0.011
GSTM1	13.128	0.419	5.6e-7	GAD2	0.275	0.014	0.011
VDAC1	31.205	50.315	5.6e-7	NAIP	28.09	57.102	0.011
MRPL51	22.034	34.018	6.1e-7	IL2RB	80.123	22.574	0.011
CACNA2D2	13.443	2.575	6.1e-7	PSMB3	91.052	123.692	0.011
CDCA2	0.158	0.705	6.8e-7	DNAH10	0.872	0.233	0.011
CTSB	159.233	426.896	7.1e-7	PTENP1	0.211	0.026	0.011
COX5A	68.791	120.535	7.2e-7	PI3	66.353	1.806	0.011
IGLV3-25	4.24	548.645	7.6e-7	ABHD17A	146.692	58.149	0.011
IGHV3-48	10.505	234.173	7.8e-7	KANK3	1.858	0.538	0.011
MRPL15	5.086	14.354	7.8e-7	SCPEP1	65.898	155.692	0.011
IGKV1-5	16.788	186.526	7.8e-7	PLA2G4A	1.094	3.244	0.011
ZMAT4	2.317	0.49	7.8e-7	ANKRD20A5P	11.713	4.105	0.011
BUB1B	1.043	2.473	8.5e-7	RHEBL1	6.273	2.115	0.011
TRIP13	0.541	1.496	9.1e-7	PTMAP4	4.824	0.255	0.011
CLEC12A	49.127	251.833	9.2e-7	PCK2	10.212	14.69	0.012
LST1	0	1.091	1.0e-6	SHMT1	4.629	9.258	0.012
C1QB	1.205	15.558	1.0e-6	DNAAF2	4.708	2.13	0.012
ECHDC3	1.621	8.348	1.1e-6	AAMP	37.783	43.966	0.012
RRM2	2.377	13.254	1.1e-6	PGK1	150.937	194.546	0.012
CAV1	0.093	0.841	1.2e-6	SERPINB10	0.776	1.846	0.012
CBWD2	20.115	29.571	1.3e-6	BCL2L13	18.152	25.365	0.012
PTGFR	0	0.571	1.3e-6	SKI	29.038	13.93	0.012
IGKV2-24	2.017	98.091	1.6e-6	HES4	5.021	1.362	0.012
C1QA	3.084	17.095	1.6e-6	SEC61A1	73.485	85.291	0.012
CAMK2N1	2.903	0.213	1.6e-6	YES1	4.496	1.583	0.012
PAPSS1	11.566	16.758	1.7e-6	VSIG10L	0.385	0.898	0.012
KLRB1	64.65	11.437	1.7e-6	GSTM2	61.324	21.042	0.012
CREG1	32.094	83.257	1.7e-6	SLC39A11	16.68	19.431	0.012
SLC1A7	4.983	0.331	1.8e-6	SAMD3	81.036	28.766	0.012
CCNB1	1.353	3.683	1.9e-6	ADAMTS1	1.432	0.422	0.012
POLQ	0.875	1.576	1.9e-6	KNSTRN	3.537	5.664	0.012
NCAPG	0.592	1.824	2.1e-6	TRIM10	0.249	0.006	0.012
NETO2	2.221	8.296	2.6e-6	M6PR	120.23	128.88	0.012
VDAC2P2	3.409	0.36	2.6e-6	SMTN	2.357	6.232	0.012
LAMA2	0.711	0.284	2.7e-6	SIGLEC9	20.605	41.152	0.012
TMTC1	0.779	3.207	2.7e-6	AC111000.1	0.809	0.011	0.012
TOPORS	11.955	3.664	2.9e-6	FPGT	3.251	5.646	0.012
TRIM51BP	2.49	0.009	3.0e-6	SGIP1	0.21	2.121	0.012
KLF15	16.434	0.076	3.0e-6	NANS	30.941	37.55	0.012
EPHX4	1.318	0.167	3.1e-6	OR7E122P	2.555	1.194	0.012
PRSS23	18.449	3.943	3.3e-6	KCNK17	1.221	0.087	0.012
IGHV5-51	6.128	7.2e-4	3.6e-6	OVCA2	15.733	20.799	0.012
AKR1C3	7.34	2.048	3.9e-6	TATDN1P1	2.061	0.223	0.012
EPS8	2.558	5.531	4.0e-6	PRR11	3.612	4.997	0.012
UGT2B11	3.611	0.044	4.1e-6	ZDHHC5	21.66	22.159	0.012

Table S1. Continuation

Gene Symbol	Base Mean Control	Base Mean COVID-19 patients	P adj	Gene Symbol	Base Mean Control	Base Mean COVID-19 patients	P adj
PSMD3	28.092	36.819	4.1e-6	PLLP	12.59	1.422	0.012
IGHV3-33	14.368	328.873	4.1e-6	NDUFB9	99.552	137.741	0.012
PRSS57	4.577	0.616	4.2e-6	PREB	18.014	23.3	0.012
AC270300.3	11.073	0	4.2e-6	ATP6V1B2	62.337	108.968	0.012
ENO1	240.435	473.846	4.4e-6	PYCARD	52.508	77.371	0.012
HLA-H	0	0.795	4.4e-6	PNP	40.426	77.881	0.012
P2RX4	22.575	37.543	4.4e-6	DDX49	23.691	25.303	0.012
MYOF	3.573	14.288	5.0e-6	STIL	1.715	1.963	0.012
LAMC1	0.595	4.114	5.0e-6	SNX19	20.72	24.74	0.012
CXCL8	1787.84	52.492	5.3e-6	MICB	0.109	0	0.012
AL162151.2	14.743	0	5.7e-6	THG1L	4.678	7.191	0.013
CBWD1	19.9	30.822	6.1e-6	C11orf74	1.818	3.804	0.013
FLVCR2	4.406	18.436	6.2e-6	DUSP2	84.114	19.859	0.013
CCR2	5.281	27.128	6.4e-6	COPG2	5.193	6.17	0.013
TIMM8B	11.654	20.935	6.4e-6	IRF5	25.71	66.691	0.013
GPX1	257.39	576.97	6.5e-6	ERBB2	7.723	3.012	0.013
PRDX1	47.926	87.357	7.5e-6	Z84492.2	7.711	2.279	0.013
ACO2	32.573	46.978	7.5e-6	LRPAP1	50.282	67.262	0.013
TROAP	1.34	2.962	8.6e-6	NOG	1.679	0.183	0.013
IGHV2-70	2.493	169.103	9.2e-6	STARD13	0.179	1.179	0.013
KLRG1	41.434	6.394	9.2e-6	RAD23B	33.274	44.127	0.013
IDH1	10.373	27.736	9.2e-6	IGKV6-21	1.131	12.446	0.013
IGHV3-30	8.137	568.55	9.7e-6	TOR4A	3.698	6.214	0.013
SOX13	9.896	2.163	9.8e-6	KIAA1147	19.994	8.397	0.013
EGR3	6.308	0.264	1.0e-5	AC089984.2	2.886	0.39	0.013
AC025884.1	7.172	0.916	1.1e-5	CDC42BPA	0.452	0.986	0.013
CST7	207.015	47.764	1.2e-5	SYNGR2	66.528	95.663	0.013
PSMA4	59.826	99.641	1.2e-5	DDIT4	59.649	123.993	0.013
PPARGC1B	3.554	5.784	1.3e-5	IGIP	1.891	0.595	0.013
KIF15	0.379	0.911	1.3e-5	HNRNPA1P76	1.236	0.146	0.013
AC114797.1	4.421	0.021	1.3e-5	GK5	39.492	15.842	0.013
C15orf38-AP3S2	1.034	4.455	1.4e-5	NBPF3	17.857	7.479	0.013
TFDP2	27.649	10.574	1.4e-5	GGH	1.535	3.722	0.013
TMIGD3	0.584	15.382	1.4e-5	NOV	2.708	0.412	0.013
IGKV1-9	0.816	74.272	1.4e-5	AC073349.2	4.362	1.65	0.013
ATP5PB	96.321	155.866	1.5e-5	FKBP1B	2.36	5.538	0.013
IGLV2-14	15.01	979.642	1.5e-5	TEX101	1.506	0.09	0.013
MSR1	6.87	15.195	1.6e-5	SERPINB1	89.085	141.268	0.013
ABCF2	1.996	4.736	1.6e-5	PLOD1	22.862	33.907	0.014
PRSS33	1.029	0.024	1.6e-5	ST6GALNAC3	1.282	4.662	0.014
EXO1	0.678	1.799	1.7e-5	EPSTI1	17.449	35.394	0.014
IGLV6-57	5.856	196.594	1.7e-5	IGLV2-8	8.539	62.889	0.014
AP2S1	48.609	80.052	1.7e-5	AL008707.1	0.022	2.167	0.014
CCDC184	1.137	0.168	1.8e-5	AL354822.1	3.322	0.822	0.014
ZBTB20	30.243	14.798	1.8e-5	HP	3.651	19.016	0.014
IGKV3-15	24.461	173.997	1.8e-5	PPP1R13B	14.466	7.776	0.014
BRD2	0.25	3.286	1.8e-5	UCHL3	13.393	18.073	0.014
GPER1	0.343	5.079	1.9e-5	PRC1	12.035	11.082	0.014
BCKDHA	15.627	33.093	1.9e-5	CABP4	4.994	1.793	0.014
HSD17B10	33.18	45.464	1.9e-5	IL10	0.703	2.357	0.014
TCN2	4.308	34.113	1.9e-5	NCR3	27.123	5.416	0.014
MMP19	0.238	2.118	2.0e-5	TMEM144	4.607	23.124	0.014

Table S1. Continuation

Gene Symbol	Base Mean Control	Base Mean COVID-19 patients	P adj	Gene Symbol	Base Mean Control	Base Mean COVID-19 patients	P adj
RPL10P19	9.824	1.449	2.1e-5	BTG2	237.153	50.273	0.014
KCNMA1	0.549	2.604	2.1e-5	EAF2	11.304	24.371	0.014
CENPU	2.785	6.911	2.1e-5	MYBPH	0.544	0.034	0.014
SLC22A16	1.447	3.717	2.2e-5	TTC22	7.317	2.436	0.014
TYMS	2.653	11.42	2.3e-5	PRPS1P2	3.694	5.416	0.014
AREG	4.217	58.781	2.3e-5	GNL2	31.322	33.186	0.014
IGLV2-11	12.38	159.532	2.3e-5	ACP2	8.891	10.756	0.014
FAM169A	5.757	1.874	2.3e-5	TNS3	6.192	14.364	0.014
ANLN	0.498	1.239	2.4e-5	HESX1	0.243	1.413	0.014
PLK1	1.139	3.825	2.4e-5	STEAP3	1.501	3.398	0.014
DDR1	2.024	0.696	2.5e-5	OGDH	34.356	39.736	0.014
IGHV3-21	14.426	206.158	2.6e-5	PDCD6IP	74.334	115.663	0.014
TPSAB1	1.408	0	2.6e-5	ARPIN	1.199	4.46	0.014
KIR2DL3	4.668	0.005	2.6e-5	OPN3	8.367	12.653	0.014
AC234301.3	0.965	38.078	2.6e-5	DPP3	19.008	21.483	0.014
METTL9	81.502	219.841	2.7e-5	AC015802.6	5.989	2.932	0.014
PTGDR	14.951	2.94	2.8e-5	HNRNPMP1	0.563	0.033	0.014
MILR1	15.846	41.773	3.3e-5	NDUFB1	88.524	145.113	0.014
RNASE4	1.899	16.235	3.4e-5	C12orf4	7.972	10.105	0.014
SCD	0.956	2.399	3.4e-5	MAFB	15.108	38.606	0.015
LINC01835	7.447	0	3.4e-5	IGKV3D-11	0.418	4.32	0.015
GLDN	0.165	4.644	3.4e-5	OSCAR	1.448	3.66	0.015
ABCD1	7.989	14.329	3.4e-5	MLLT6	189.545	82.789	0.015
UAP1L1	8.971	19.184	3.4e-5	GIMAP3P	2.723	0.893	0.015
CENPF	2.287	2.779	3.4e-5	S100A6	543.902	814.268	0.015
KCTD3	3.881	8.349	3.4e-5	DMAC2	17.879	22.287	0.015
CDC6	0.676	1.741	3.4e-5	CXCL5	7.401	0.533	0.015
NRG1	3.004	11.141	3.4e-5	BCKDK	13.747	22.597	0.015
ESPL1	0.624	1.468	3.5e-5	AC022217.1	0.66	5.526	0.015
CLDN10	3.605	0.52	3.7e-5	CLDN22	0.344	0	0.015
FGF23	19.728	0.024	3.9e-5	AC138409.1	1.751	0.288	0.015
CANX	49.499	104.921	3.9e-5	ARHGEF25	0.905	0.407	0.015
PLD3	95.003	170.321	3.9e-5	KDELR1	35.721	42.848	0.015
LGR6	13.14	2.422	4.0e-5	PCCB	10.21	11.623	0.015
ALOX15B	0.487	14.507	4.2e-5	ITGB8	1.204	0.188	0.015
SH3PXD2B	0.374	2.275	4.3e-5	MRPS10	8.881	10.204	0.015
SEMA4C	19.178	4.637	4.4e-5	PSTPIP2	25.602	42.767	0.015
CDK5	4.916	10.277	4.6e-5	SRPK2	49.594	23.554	0.015
CD163	34.924	315.372	4.6e-5	ARID4B	64.52	19.747	0.015
CEP78	49.569	13.335	4.7e-5	GTF3C6	21.073	25.075	0.015
ZBTB43	12.386	22.674	4.8e-5	SQLE	8.065	9.825	0.015
RAPH1	0.847	1.456	4.9e-5	UQCRFS1P1	9.84	13.329	0.015
SDC1	0.036	1.687	4.9e-5	C12orf29	10.151	5.7	0.015
TCN1	4.821	0.434	4.9e-5	LPCAT3	17.363	17.051	0.015
PSMB6	54.917	73.01	4.9e-5	TMEM256	33.693	47.673	0.015
COPB2	26.015	34.545	4.9e-5	PIGY	6.779	1.8e-5	0.015
LINC00869	311.913	106.34	5.0e-5	DHRS3	15.644	4.308	0.016
ATP5MF	151.497	236.712	5.0e-5	EMILIN2	24.279	48.873	0.016
IGHV1-18	13.275	122.318	5.1e-5	MEF2A	25.855	41.393	0.016
TNFSF10	24.927	44.715	5.1e-5	FGFBP2	197.209	41.852	0.016
CDC45	0.568	2.516	5.2e-5	MYD88	64.214	105.784	0.016
CBX7	53.965	22.095	5.3e-5	IGFBP3	4.099	0.4	0.016

Table S1. Continuation

Gene Symbol	Base Mean Control	Base Mean COVID-19 patients	P adj	Gene Symbol	Base Mean Control	Base Mean COVID-19 patients	P adj
ADGRB2	1.484	0.313	5.3e-5	PRDM5	13.221	5.549	0.016
WDR18	9.393	12.629	5.4e-5	SDCCAG8	13.377	19.009	0.016
PZP	12.203	1.253	5.5e-5	EPHB2	0.923	4.624	0.016
SYTL2	98.155	15.614	5.5e-5	TSG101	37.041	37.981	0.016
GBGT1	8.596	16.679	5.6e-5	KIAA2026	37.401	20.084	0.016
NLRP2	0	0.245	5.6e-5	TOLLIP	25.221	36.338	0.016
FAM96A	32.643	48.593	5.7e-5	ZC3H12C	0.431	1.246	0.016
HCG4P7	0.119	1.488	5.7e-5	RPUSD2	7.813	3.107	0.016
DEPDC1	0.143	0.318	5.8e-5	IGHV3-33	5.709	526.69	0.016
IGHV3-53	0.694	24.312	5.8e-5	CDKN2B	1.515	5.992	0.016
PRRG3	1.524	0.029	6.2e-5	TSEN54	66.477	25.637	0.016
MYO1E	3.516	8.932	6.3e-5	FAM212A	1.133	4.446	0.016
ZFPM1	10.771	2.689	6.3e-5	IFITM3	92.511	237.04	0.016
MINOS1-NBL1	0	0.668	6.4e-5	ZNF611	26.542	15.393	0.016
PRDX3	23.048	49.617	6.5e-5	FGF14	0.085	5.5e-4	0.016
IGLV1-47	17.822	162.412	6.6e-5	GYS1	29.258	26.385	0.016
ANKRD30BL	85.39	1.359	6.8e-5	PSMD14	14.155	14.839	0.016
TIMP4	0.005	1.757	7.0e-5	C8G	7.85	2.549	0.016
RAB20	3.254	11.783	7.0e-5	MUC6	0.827	0.149	0.016
DHCR24	3.141	4.94	7.0e-5	ANKRD20A7P	0.367	0.039	0.016
TNFAIP8L2	5.129	10.364	7.1e-5	TRMT6	7.977	18.428	0.016
IGHV3-11	4.661	72.072	7.1e-5	CNDP2	77.152	99.392	0.016
NCALD	22.922	7.241	7.3e-5	DNAH10	1.32	0.844	0.016
NTSR1	0.731	7.257	7.7e-5	YRDC	19.215	7.937	0.016
MT1E	1.391	5.044	7.8e-5	SASH1	1.306	3.225	0.016
TSPOAP1	61.57	23.496	7.8e-5	PODN	1.243	0.327	0.016
PSMB2	18.235	26.967	8.0e-5	CBX2	0.08	0.454	0.016
NME2	136.872	188.112	8.2e-5	DCTN6	17.034	20.412	0.016
IGHG2	24.574	130.91	8.6e-5	CKB	5.858	1.768	0.016
IGLV4-69	6.499	66.624	8.6e-5	PLPP3	0.23	0.88	0.016
COLGALT2	2.748	0.527	8.6e-5	AHCY	13.965	18.777	0.016
EZH2	13.902	26.517	8.6e-5	HLA-V	1.343	0.075	0.016
LTBP4	86.289	22.204	8.6e-5	RNF144A	9.593	3.333	0.016
IGHV6-1	5.071	54.68	8.6e-5	AC006509.1	5.925	0.601	0.016
MICU1	16.862	27.969	8.6e-5	JADE1	19.578	10.051	0.017
CAPRIN1	54.022	61.695	8.6e-5	FUT11	22.387	8.646	0.017
IGKC	351.426	3416.79	8.7e-5	TRIM27	11.661	9.696	0.017
PTCH1	20.192	7.148	8.7e-5	H2AFY	142.189	279.604	0.017
VSTM2B	0.591	0.038	8.8e-5	ATG101	13.696	14.71	0.017
SPTBN5	4.564	1.477	8.8e-5	HDHD5	13.66	22.204	0.017
B3GAT1	20.555	3.977	8.9e-5	ITGA7	1.041	2.342	0.017
LYPD2	2.812	0.014	8.9e-5	ITFG1	17.503	20.9	0.017
IGKV1-17	1.488	43.254	9.0e-5	OXA1L	105.83	131.73	0.017
SLC43A3	15.805	36.044	9.0e-5	SPOCK2	132.372	33.692	0.017
PTGDR2	1.433	0.113	9.1e-5	COMT	43.619	63.133	0.017
IGKV2D-29	3.2	148.455	9.2e-5	IGHV3-72	0.842	6.346	0.017
RENBP	15.62	24.883	9.2e-5	RAD54L	1.599	1.696	0.017
KRT17P8	10.48	0.958	9.3e-5	TMTC3	3.856	2.243	0.017
ANO5	0.141	0.612	9.6e-5	BST2	80.496	103.199	0.017
UPK3B	2.246	0.51	9.6e-5	MTHFR	30.731	34.448	0.017
AKT3	16.211	5.574	1.0e-4	DUSP16	13.966	5.345	0.017

Table S1. Continuation

Gene Symbol	Base Mean Control	Base Mean COVID-19 patients	P adj	Gene Symbol	Base Mean Control	Base Mean COVID-19 patients	P adj
TGFBR3	38.138	6.538	1.0e-4	TM4SF19-TCTEX1D2	1.651	0.337	0.017
CKS2	4.062	9.387	1.0e-4	HVCN1	29.201	48.233	0.017
LAP3	30.484	60.4	1.1e-4	JAGN1	11.254	14.32	0.017
ACVR1B	5.833	14.812	1.1e-4	AC245052.2	14.446	2.318	0.017
CEP55	0.417	1.389	1.1e-4	FGD3	113.37	59.836	0.017
GNPDA1	9.484	18.113	1.1e-4	TMEM115	7.094	7.741	0.017
C1orf43	59.021	88.785	1.1e-4	TRAJ37	38.606	4.362	0.017
CHRFAM7A	0	0.238	1.1e-4	LRIG1	11.833	3.313	0.017
ETHE1	20.773	25.917	1.2e-4	HLA-V	14.11	0.764	0.017
IGLV7-43	3.527	22.958	1.2e-4	CHSY3	0.613	0.014	0.017
PBK	0.041	0.712	1.2e-4	PNKD	32.826	54.406	0.018
MAGEE1	3.074	0.578	1.2e-4	AP001885.1	3.683	0.963	0.018
ERAL1	11.526	13.841	1.3e-4	ANXA4	23.036	36.092	0.018
PPARG	0.813	6.795	1.3e-4	ITSN1	5.273	13.221	0.018
IGLV3-1	13.422	521.275	1.3e-4	CFL1	594.985	627.124	0.018
UBE2C	1.285	6.468	1.3e-4	CDH2	0.135	0.008	0.018
IGLV1-51	26.325	1199.347	1.4e-4	KCNE1	3.953	11.858	0.018
SAPCD2	1.289	4.477	1.4e-4	RB1	22.177	20.892	0.018
AKAP12	1.013	0.045	1.4e-4	PRDM8	8.889	3.772	0.018
FLT3	2.67	42.322	1.4e-4	GPM6B	3.057	1.231	0.018
CYC1	47.124	65.141	1.5e-4	LGALS9C	7.823	2.433	0.018
AGK	6.287	4.165	1.5e-4	SCT	5.754	0.794	0.018
CA8	2.154	0.174	1.6e-4	CLTCL1	1.93	6.329	0.018
KIF18B	0.31	1.061	1.6e-4	UNC5A	0.284	0.665	0.018
RPS6KA5	14.172	4.525	1.6e-4	ARHGAP23	0.185	0.577	0.018
LDHA	134.008	238.669	1.6e-4	AC010240.2	2.251	0.438	0.018
CDCA3	1.147	3.005	1.6e-4	COLGALT1	44.51	62.037	0.018
CAT	43.231	78.819	1.6e-4	CLIC5	1.553	0.395	0.018
PDIA5	3.215	7.921	1.6e-4	OXR1	16.642	19.054	0.018
SNAPIN	11.54	20.688	1.6e-4	FYN	304.225	136.676	0.018
SULT1A1	19.488	67.051	1.6e-4	TMEM205	34.318	52.844	0.018
USH1G	0.774	0.127	1.6e-4	ISM1	0.891	0.113	0.018
AC245100.4	17.444	6.148	1.7e-4	MARC1	15.868	71.543	0.018
IGHV3-13	1.866	67.81	1.7e-4	DLC1	0.043	0.245	0.018
KIF23	1.817	2.778	1.7e-4	ATP5PF	50.16	63.462	0.018
PDGFD	4.493	1.202	1.7e-4	PLBD1	99.069	330.165	0.018
CD163L1	0.184	2.161	1.7e-4	HMOX1	28.871	59.696	0.018
HPRT1	13.265	16.711	1.7e-4	AMMECR1	9.746	4.822	0.018
MYO6	2.021	0.56	1.7e-4	NLRC4	2.643	5.789	0.018
EMC8	14.602	17.076	1.8e-4	DCPS	9.737	15.219	0.018
TPI1	143.603	197.113	1.8e-4	JAG1	2.517	5.785	0.018
LSS	19.425	7.989	1.8e-4	CEBPA	12.456	22.515	0.018
RHOBTB3	7.186	2.956	1.8e-4	ECHDC1	25.644	36.183	0.018
NCAPH	0.713	2.426	1.8e-4	TMEM37	0	0.225	0.018
IGHV3-23	57.624	389.158	1.8e-4	FRMPD3	2.365	0.542	0.018
CD247	212.6	56.193	1.9e-4	LSM6	26.398	36.917	0.018
OLFML2A	0.226	2.149	1.9e-4	ZXDB	9.071	3.6	0.018
E2F8	0.105	0.713	1.9e-4	GAS2L3	1.189	2.75	0.018
RPL23AP23	5.339	0.627	1.9e-4	RAD51AP1	0.78	2.196	0.018
MTCH2	21.962	35.06	1.9e-4	MSANTD3	10.414	4.048	0.018
SKA3	0.157	0.701	1.9e-4	GFRA2	0.777	1.383	0.018

Table S1. Continuation

Gene Symbol	Base Mean Control	Base Mean COVID-19 patients	P adj	Gene Symbol	Base Mean Control	Base Mean COVID-19 patients	P adj
PSMA1	85.417	91.808	2.0e-4	TGM3	0.445	0.021	0.018
JCHAIN	51.873	649.498	2.0e-4	IGHG3	1.657	21.123	0.019
EEF1AKMT4	0.509	1.389	2.0e-4	GIMAP8	8.17	11.574	0.019
POMP	22.789	38.113	2.0e-4	ADGRE3	19.059	1.359	0.019
TXNDC5	17.491	144.425	2.1e-4	H1FX	120.755	157.523	0.019
CAMP	8.114	2.003	2.1e-4	KCTD12	18.731	49.907	0.019
IGKV1-12	6.538	76.282	2.2e-4	C17orf107	2.608	0.544	0.019
HMMR	0.436	0.995	2.2e-4	DECR1	82.616	85.782	0.019
IGLV1-40	23.133	199.172	2.2e-4	ATP6V0A1	32.689	46.897	0.019
UTP4	4.081	1.076	2.3e-4	MUC6	1.561	0.422	0.019
IGHV2-5	9.698	178.482	2.3e-4	RSBN1	17.946	9.159	0.019
SIGLEC12	0.024	0.863	2.3e-4	SH3TC1	26.327	43.325	0.019
AK4	0.801	1.457	2.3e-4	KIF21B	36.339	12.97	0.019
SMIM15	6.013	10.92	2.3e-4	ARL10	11.715	9.395	0.019
NRXN2	0.739	0.078	2.4e-4	SND1	44.161	50.212	0.019
GLI1	5.371	1.794	2.4e-4	PDGFC	1.35	3.128	0.019
IGHV4-59	13.432	292.372	2.4e-4	FLOT1	17.044	4.781	0.019
CCSER2	30.537	16.238	2.4e-4	ODF3	0.359	0.101	0.019
ULK4P1	0.943	4.097	2.5e-4	MRPL36	11.324	17.538	0.019
IGKV3-11	30.006	440.476	2.5e-4	ARHGEF11	16.491	24.819	0.019
EPPK1	1.006	0.067	2.6e-4	HLA-DRA	0.042	1.465	0.019
EDARADD	2.113	0.473	2.7e-4	IGHGP	0.126	1.158	0.019
MAML1	7.321	9.598	2.7e-4	COX6A1	147.38	182.831	0.019
GALK1	9.636	17.642	2.8e-4	CSRP2	2.42	0.578	0.019
NDUFA7	25.592	40.209	2.9e-4	AGAP12P	1.01	0.365	0.019
RGS16	0.321	1.098	2.9e-4	SYTL5	0.05	0	0.019
TMED9	52.443	65.208	2.9e-4	SNTB1	13.527	16.444	0.019
SDHD	35.972	62.545	2.9e-4	KLRF1	71.087	18.956	0.019
CHRFAM7A	1.853	6.04	2.9e-4	ABI2	14.704	8.169	0.019
SLC39A1	14.504	19.651	2.9e-4	CKAP2L	0.444	0.67	0.019
YWHAE	0.198	4.563	3.0e-4	RPS27AP7	9.272	1.767	0.019
CLEC12B	6.295	33.52	3.1e-4	DAAM2	0.437	11.598	0.019
LAMTOR2	29.347	39.836	3.1e-4	FBN2	1.453	5.445	0.019
GAPDH	567.198	1392.836	3.1e-4	HTR7P1	0.732	1.02	0.02
SMIM10	0.137	0.728	3.1e-4	KIF19	3.729	0.7	0.02
KIF2C	0.7	1.972	3.2e-4	SLC9A3R1	143.21	52.958	0.02
ZFYVE28	34.109	8.595	3.2e-4	AL049757.1	0.576	0.122	0.02
ZNF365	4.749	0.806	3.2e-4	AGFG1	38.268	94.497	0.02
PTPRO	1.96	3.57	3.2e-4	FGFR1OP	28.664	10.716	0.02
RAB13	5.22	18.117	3.3e-4	IGHV2-70D	0	2.526	0.02
ENPP5	2.434	0.624	3.3e-4	MICD	0.002	0.283	0.02
GRB10	6.375	30.667	3.3e-4	CNTNAP1	9.604	4.787	0.02
EFNA5	2.01	0.204	3.3e-4	SYK	40.607	75.105	0.02
OCRL	2.503	4.709	3.3e-4	CCNJ	5.169	6.157	0.02
NUBP1	15.046	21.749	3.4e-4	TMEM64	6.09	2.575	0.02
TTK	0.335	1.167	3.4e-4	UBE2L3	20.591	24.923	0.02
ARL4D	1.683	0.403	3.4e-4	ORC1	0.83	1.275	0.02
FRRS1	1.046	3.021	3.5e-4	BOD1L1	47.153	26.214	0.02
MAFG	9.305	19.09	3.5e-4	FBXL14	13.402	5.486	0.02
IGLC2	115.388	1972.762	3.6e-4	ANKRD36	18.738	6.793	0.02
PLEKHN1	0.355	0.874	3.6e-4	AL353662.1	8.566	0.679	0.02
ZWINT	0.835	4.267	3.6e-4	NEURL1	9.285	3.147	0.02

Table S1. Continuation

Gene Symbol	Base Mean Control	Base Mean COVID-19 patients	P adj	Gene Symbol	Base Mean Control	Base Mean COVID-19 patients	P adj
NKIRAS2	22.337	33.863	3.7e-4	AMOT	1.179	0.369	0.02
CDHR3	6.743	1.856	3.7e-4	EIF3I	76.205	79.877	0.02
MRPL53	18.297	37.785	3.7e-4	AAK1	82.221	37.492	0.02
DTL	0.255	1.79	3.7e-4	GATA2	17.607	1.577	0.02
NDUFAB1	23.115	33.819	3.9e-4	JADE2	49.552	21.799	0.02
GSTZ1	4.052	9.063	3.9e-4	PRELID1	83.98	126.277	0.02
TRDJ1	236.614	43.398	3.9e-4	SLC37A2	8.302	16.315	0.02
TMED7-TICAM2	1.469	2.548	4.1e-4	AGPAT3	36.136	44.896	0.02
IGKV1-33	12.041	290.495	4.1e-4	PIP5K1B	2.651	4.652	0.021
IGLL5	8.923	94.584	4.2e-4	ZBTB4	24.651	10.361	0.021
OAS1	30.022	87.206	4.2e-4	WNT10B	8.264	1.71	0.021
SIGLEC1	2.601	33.632	4.2e-4	CYP46A1	3.028	0.773	0.021
FAM72B	0.893	1.847	4.2e-4	CYTH1	233.83	112.474	0.021
PPT1	90.615	180.605	4.2e-4	HOMER1	0.498	0.201	0.021
CLDND2	26.719	6.855	4.3e-4	GNS	39.645	65.832	0.021
TMEM106A	7.846	14.455	4.4e-4	FCGR3A	195.521	56.354	0.021
ERLIN1	5.133	14.83	4.4e-4	TRGV9	3.726	1.193	0.021
PSMB7	74.346	108.318	4.6e-4	RCAN1	5.766	7.807	0.021
LHFPL2	5.233	16.222	4.6e-4	C9orf131	6.864	4.2	0.021
IGHG3	2.634	46.989	4.7e-4	NDUFV2	75.068	80.012	0.021
CYBRD1	11.899	20.996	4.7e-4	NSF	11.947	14.68	0.021
AC010970.1	24524.215	88.265	4.7e-4	MRPS18A	11.945	14.353	0.021
PSMD8	57.746	75.229	4.7e-4	TNFRSF10C	89.75	7.682	0.021
SLC16A14	0.125	0.544	4.8e-4	C10orf76	15.543	17.085	0.021
NAP1L2	1.382	0.291	4.8e-4	C16orf70	20.272	25.677	0.021
CIT	1.035	1.688	4.9e-4	HAVCR2	8.972	14.11	0.021
FAM129B	11.306	26.778	4.9e-4	PSMG2	34.37	38.765	0.021
YIF1A	17.84	21.283	4.9e-4	TMEM45B	5.086	1.201	0.021
LTA4H	84.631	165.748	5.0e-4	PAK6	2.185	0.739	0.021
CEP126	2.396	0.796	5.0e-4	CXCL1	9.774	0.798	0.021
TSPO	133.622	226.939	5.0e-4	C14orf28	2.178	0.978	0.021
ATP6V1F	87.921	133.048	5.0e-4	LHFPL6	0.204	1.101	0.021
NDUFV3	11.205	20.101	5.1e-4	AK5	14.058	4.04	0.021
AL365475.1	1.762	0.418	5.1e-4	ZNF213	3.443	4.41	0.021
RUNX3	178.694	49.61	5.1e-4	KLKB1	1.672	0.396	0.021
RPL9P9	40.697	6.401	5.1e-4	GBA	19.521	25.599	0.021
AC098679.2	0.769	2.722	5.1e-4	NFS1	7.379	9.422	0.021
G0S2	1159.159	59.155	5.1e-4	TPT1P12	4.62	0.618	0.021
CDK17	40.015	15.95	5.2e-4	TMEM70	8.863	18.861	0.021
TRGV4	23.05	6.311	5.2e-4	PREP	13.351	16.187	0.021
MGAT1	97.004	153.232	5.2e-4	PCMT1	31.372	39.135	0.021
ABCA5	26.172	11.283	5.2e-4	BAHCC1	1.452	4.357	0.021
CCL4	2.051	0.026	5.3e-4	MRPL58	8.911	13.92	0.021
IGLV3-10	4.443	200.73	5.3e-4	C21orf2	33.163	14.886	0.021
SLC35F3	0.43	0.062	5.5e-4	FEZ1	5.419	1.749	0.021
MDH2	52.356	57.565	5.5e-4	ARHGAP23	0.017	0.169	0.021
CCT5	60.45	93.661	5.5e-4	GSTA1	1.457	0.026	0.021
AC243791.2	6.025	1.97	5.5e-4	ICAM4	5.456	8.114	0.021
GHRL	9.462	3.346	5.5e-4	PLA2G6	68.403	29.461	0.021
TUBG1	5.491	9.095	5.6e-4	TBC1D19	3.513	1.082	0.021
NDUFS6	55.382	71.859	5.6e-4	NDUFS2	57.245	61.952	0.022
SORBS2	0.794	0.129	5.7e-4	LRRC8D	18.056	26.585	0.022

Table S1. Continuation

Gene Symbol	Base Mean Control	Base Mean COVID-19 patients	P adj	Gene Symbol	Base Mean Control	Base Mean COVID-19 patients	P adj
NEUROD2	8.862	0.041	5.8e-4	THUMPD1	48.769	24.089	0.022
YWHAG	9.309	20.611	5.8e-4	AL031281.1	9.601	2.134	0.022
DOCK1	0.167	1.024	5.8e-4	POU3F2	0.101	0.001	0.022
RBFOX2	0.516	0.084	5.8e-4	SIRPD	2.242	8.047	0.022
ZNF366	0.609	2.899	5.8e-4	GAS6	5.604	7.403	0.022
KRT5	1.118	0.104	5.9e-4	BCL9L	88.114	26.879	0.022
AK2	46.211	65.948	5.9e-4	TMEM268	11.017	12.943	0.022
RPL26L1	9.439	16.039	6.0e-4	TXNDC11	31.018	35.683	0.022
CCNA2	1.153	2.416	6.2e-4	THSD7A	0.046	0.023	0.022
CORO6	2.417	0.689	6.2e-4	FAT2	0.279	0.377	0.022
PLEKHA1	23.337	9.759	6.4e-4	HMGCL	13.151	14.34	0.022
MLF1	5.527	1.698	6.4e-4	PFN2	3.169	1.291	0.022
TPST1	6.259	45.959	6.4e-4	SSR1	45.852	68.427	0.022
IGLV3-19	18.587	111.861	6.4e-4	IGHV1-46	3.702	46.692	0.022
IGLV9-49	1.214	17.696	6.4e-4	PTRHD1	14.563	19.264	0.022
LRRC43	2.802	0.632	6.5e-4	TKTL1	9.963	1.508	0.022
HDLBP	74.087	108.822	6.5e-4	DNAJC15	4.506	9.074	0.022
AL359976.1	1.238	0.013	6.5e-4	RCC2	25.588	28.333	0.022
SHROOM1	1.124	7.057	6.5e-4	CXorf65	29.62	12.668	0.022
KIAA0930	31.821	55.978	6.6e-4	SLIRP	37.4	55.846	0.022
DMPK	23.148	8.847	6.6e-4	KAT6B	12.965	5.583	0.022
SPAG5	7.855	11.063	6.6e-4	FAM35BP	0.222	0.594	0.022
EEF1A1P3	2.721	0.366	6.7e-4	DEPP1	2.58	6.058	0.022
NEO1	3.346	1.78	6.8e-4	ADAM23	1.965	0.639	0.022
CNOT6L	47.983	23.057	6.8e-4	HLA-DPB2	0.497	0.01	0.022
XIAPP3	6.773	1.439	6.8e-4	CCT6A	44.79	51.272	0.023
NDUFAF3	23.027	31.789	6.8e-4	ASNA1	35.905	48.259	0.023
NUF2	1.231	3.71	6.9e-4	CTNND1	18.523	16.668	0.023
A2MP1	9.116	1.14	6.9e-4	SLC25A5	119.353	167.841	0.023
ORMDL2	12.916	16.2	6.9e-4	TMIE	0.624	0.1	0.023
ALOX15	1.057	0.092	6.9e-4	LAIR1	7.142	16.117	0.023
RRAS	13.784	20.881	6.9e-4	IGHV4-4	4.65	45.86	0.023
NDUFB5	26.726	43.303	7.0e-4	WWC2	1.836	3.044	0.023
VPS25	20.628	25.9	7.0e-4	CTNNA1	45.401	55.944	0.023
KMT2E	90.188	45.342	7.0e-4	SRGAP2	35.145	64.624	0.023
C1orf21	22.397	4.973	7.0e-4	SPEF2	7.965	2.65	0.023
ST14	7.23	27.182	7.0e-4	MCM2	2.637	4.922	0.023
E2F7	0.218	0.371	7.0e-4	UBQLN1	59.652	70.287	0.023
AL161672.1	1.322	0.038	7.2e-4	BFSP1	2.297	0.538	0.023
UQCRC1	84.316	99.412	7.2e-4	SLC27A3	24.91	37.129	0.023
IL18	6.095	23.818	7.4e-4	SKAP1	57.487	15.804	0.023
YIF1B	29.598	44.26	7.7e-4	GEMIN7	19.203	30.588	0.023
AC117382.1	2.605	0.145	7.7e-4	DENND1A	19.912	26.296	0.023
HADHA	64.038	92.274	7.8e-4	MAF	17.983	4.6	0.023
SYN2	0.035	0.749	7.8e-4	SLC2A9	7.745	12.806	0.023
AUTS2	33.762	8.024	7.8e-4	IGHG4	1.465	19.654	0.023
AC008750.8	6.465	1.522	8.1e-4	TEDC2	0.144	0.726	0.023
SCARB2	20.233	33.032	8.1e-4	CCDC171	1.982	0.833	0.023
SLC4A4	1.377	0.315	8.2e-4	CD40LG	7.373	2.258	0.023
DUSP7	6.129	9.024	8.3e-4	DYM	26.036	29.356	0.023
PSMA6	84.267	132.371	8.3e-4	RXFP2	0.404	1.971	0.023
PEPD	25.481	37.453	8.3e-4	ZNF100	7.263	8.856	0.023

Table S1. Continuation

Gene Symbol	Base Mean Control	Base Mean COVID-19 patients	P adj	Gene Symbol	Base Mean Control	Base Mean COVID-19 patients	P adj
PGM2	9.843	18.392	8.3e-4	HTN1	2.164	0.896	0.023
BCAT1	2.178	5.867	8.3e-4	ATP5F1A	116.061	143.302	0.023
CERS6	5.204	8.893	8.3e-4	CD101	13.095	13.947	0.023
NLN	3.727	7.601	8.4e-4	FAT4	0.295	0.057	0.023
XXYLT1	5.093	7.149	8.4e-4	HCAR2	30.06	2	0.023
ICAM5	0.393	1.227	8.5e-4	YPEL4	0.844	2.811	0.023
FAM131B	1.091	0.322	8.5e-4	ME2	28.175	33.053	0.023
ZNF831	9.935	2.333	8.5e-4	FSTL3	3.9	7.028	0.023
FZD5	0.235	0.693	8.7e-4	ASNS	28.924	11.96	0.023
AIMP2	4.679	8.535	8.9e-4	SARDH	2.412	0.593	0.023
SEC11A	70.835	108.731	8.9e-4	TRAFD1	19.781	21.047	0.024
PRDM1	23.934	36.355	8.9e-4	RASSF7	15.992	4.734	0.024
IGLV10-54	2.312	30.102	8.9e-4	CXCR1	36.322	2.708	0.024
GLMP	17.308	24.5	8.9e-4	XRCC4	2.97	4.595	0.024
TRAV10	8.107	1.245	9.0e-4	KIF5C	1.449	0.486	0.024
BMI1	59.111	27.816	9.1e-4	TPBGL	0.241	0.042	0.024
FGF9	0.891	0.357	9.1e-4	PRDM9	1.956	0.444	0.024
PKMYT1	1.244	3.729	9.1e-4	TTC9	5.304	1.813	0.024
ANXA2	156.842	354.216	9.2e-4	ATP6V1E1	41.605	51.266	0.024
TOMM22	27.733	37.897	9.4e-4	GPR183	49.381	20.829	0.024
ATP5MC3	56.352	91.447	9.8e-4	MPV17	48.413	58.088	0.024
HTRA1	0.947	11.344	9.8e-4	SCAND2P	10.819	9.321	0.024
PHF24	0.365	0.037	0.001	TPCN2	11.486	17.021	0.024
ROGDI	19.782	38.15	0.001	NMUR1	11.494	3.111	0.024
ATXN10	34.742	37.797	0.001	MYO7A	2.491	9.024	0.024
COPZ1	49.878	75.115	0.001	PURA	10.278	3.678	0.024
C16orf54	87.857	28.771	0.001	HDAC9	9.826	29.919	0.024
PKM	240.791	390.282	0.001	LMAN2	80.08	99.904	0.024
SLC38A7	6.689	10.325	0.001	MYO3B	0.758	0.181	0.024
CXCL10	0.601	5.724	0.001	NRM	12.888	17.475	0.024
NPM1P40	3.987	0.094	0.001	PSMD5	19.554	17.873	0.024
HNRNPA1P34	1.68	0.111	0.001	FBXO33	36.4	14.31	0.024
RASGEF1B	15.391	26.657	0.001	IGLV8-61	0.545	61.747	0.024
ELOVL3	0.138	0.946	0.001	TMEM63C	2.712	0.653	0.024
CLTA	30.761	51.674	0.001	DUSP3	10.86	18.586	0.024
SUCLG1	44.244	48.778	0.001	HLX	5.116	12.932	0.024
GATA3	35.821	6.742	0.001	DCTN2	64.352	66.697	0.024
GSTO1	47.353	84.371	0.001	CD1D	11.419	32.143	0.025
SELENON	13.33	15.612	0.001	SLC3A2	74.843	90.429	0.025
AGAP7P	7.04	0.661	0.001	AGBL3	7.33	1.405	0.025
S1PR5	35.304	7.924	0.001	HAUS1	12.312	14.689	0.025
TMEM208	15.336	20.647	0.001	USP51	0.447	0.124	0.025
IGKV1-27	6.108	49.152	0.001	SERPINB9	15.129	16.481	0.025
ZNF574	6.935	2.654	0.001	RNASE6	34.212	114.772	0.025
SYNE3	13.145	18.006	0.001	MGST2	12.919	27.321	0.025
BATF2	0.303	1.083	0.001	CD300C	9.615	22.551	0.025
AMPH	1.919	11.111	0.001	SMIM27	22.11	11.652	0.025
IL7R	144.8	47.629	0.001	RAB10	30.871	45.439	0.025
KCNQ1	14.706	24.634	0.001	EID3	5.052	1.665	0.025
UACA	1.938	8.319	0.001	PTPN4	53.234	23.958	0.025
MERTK	1.877	7.45	0.001	TUBB4A	3.009	0.788	0.025
ZNF830	11.305	3.381	0.001	TM9SF1	9.467	11.393	0.025

Table S1. Continuation

Gene Symbol	Base Mean Control	Base Mean COVID-19 patients	P adj	Gene Symbol	Base Mean Control	Base Mean COVID-19 patients	P adj
AMZ2P2	1.673	0.094	0.001	CAPN2	165.42	169.671	0.025
CCR9	3.653	0.76	0.001	IRAK3	19.739	64.447	0.025
RNH1	77.042	100.76	0.001	SLFN12	8.507	13.524	0.026
AC244157.2	1.618	0.101	0.001	ZCCHC18	6.671	2.395	0.026
HLA-DRB4	0.075	128.485	0.001	NDUFA8	10.079	17.384	0.026
IGHV4-34	16.724	135.488	0.001	GAS1	0.418	0.052	0.026
SPRY2	0.566	6.215	0.001	CD34	0.386	0.085	0.026
CDKN3	1.871	3.732	0.001	PGBD2	5.465	2.884	0.026
HOXA9	0.308	1.767	0.001	C20orf27	18.178	32.316	0.026
IGLV3-21	7.229	151.026	0.001	MT1JP	0.213	0.422	0.026
SQOR	29.501	52.781	0.001	MYL5	13.758	18.597	0.026
HLA-DQB1	0.048	17.076	0.001	H2AFJ	30.037	55.999	0.026
TMTC2	3.549	9.376	0.001	EXPH5	0.665	0.252	0.026
PSMA7	125.462	161.044	0.001	MPG	19.643	20.799	0.026
AC090519.1	1.392	0.163	0.001	NIFKP6	4.103	0.758	0.026
RPL7P32	3.042	0.262	0.001	TALDO1	170.304	269.245	0.026
AURKA	0.849	1.468	0.001	SPAG17	0.348	1.033	0.026
ACTG1	691.362	963.251	0.001	DEPDC1B	2.126	1.916	0.026
EIF4G1	84.261	107.042	0.001	WNT1	3.347	0.772	0.026
IGHV3-15	11.858	167.601	0.001	DNAH8	0.081	0.306	0.026
NECTIN2	2.541	7.372	0.001	TOR3A	27.821	31.002	0.026
TMEM38A	1.264	4.537	0.001	ACSS2	29.923	33.27	0.026
A2M	48.706	2.874	0.001	AC244394.1	0.67	0.083	0.026
KIF21A	9.815	2.08	0.001	SCAMP2	47.258	53.976	0.026
ARL4C	119.758	30.236	0.001	NIPA2	24.541	27.037	0.026
CXCR2	129.932	6.956	0.001	C17orf58	1.096	1.682	0.026
SH2D2A	42.047	9.538	0.001	RAPSN	1.634	0.515	0.026
TBC1D2	7.434	20.741	0.001	AC242308.2	2.505	0.807	0.026
FH	9.9	16.342	0.001	SIAH2	23.127	81.109	0.026
CARD6	1.954	3.085	0.001	MFGE8	27.009	92.185	0.026
FLOT1	0.293	3.151	0.001	HM13	102.188	108.014	0.027
FIBP	25.219	31.65	0.001	LILRB4	2.83	10.238	0.027
MAP3K6	8.049	8.474	0.002	ARHGAP24	7.257	21.836	0.027
NAA20	24.26	28.609	0.002	LILRA2	1.634	4.381	0.027
AKR1A1	38.189	52.666	0.002	GZMM	107.07	26.692	0.027
COX8A	193.042	271.74	0.002	SPNS1	37.488	38.66	0.027
LIM2	4.986	0.691	0.002	MAP1LC3B2	13.116	1.772	0.027
TP53I3	5.605	14.735	0.002	SPATS2L	4.369	7.475	0.027
ESRP2	5.805	0.864	0.002	KANSL1	19.054	20.663	0.027
AJAP1	0.259	0.035	0.002	LAIR1	1.617	24.691	0.027
MRPL18	16.541	23.51	0.002	NLGN3	2.499	0.92	0.027
TOB1	120.456	53.598	0.002	AP1S1	8.322	11.843	0.027
NDUFAF1	4.155	7.735	0.002	IDE	12.108	13.207	0.027
ACAA2	22.063	27.381	0.002	FOXP1	65.649	34.503	0.027
FCRL6	93.894	18.711	0.002	TSPAN2	19.822	5.488	0.027
IGHV4-28	0.484	10.01	0.002	DOK1	13.915	19.676	0.027
IGHV3-49	7.152	100.193	0.002	COX5B	102.564	111.67	0.027
VAMP8	112.79	151.03	0.002	MAP2K1	32.799	52.598	0.027
MZB1	8.388	89.416	0.002	AHCYL1	18.845	22.572	0.027
CLDN24	1.212	0.055	0.002	LDLRAD3	2.104	7.369	0.028
OTOF	0.816	6.768	0.002	C1orf146	1.255	0.34	0.028
CDCA8	0.801	1.624	0.002	CTSA	109.974	202.268	0.028

Table S1. Continuation

Gene Symbol	Base Mean Control	Base Mean COVID-19 patients	P adj	Gene Symbol	Base Mean Control	Base Mean COVID-19 patients	P adj
B4GALNT3	0.862	0.221	0.002	CYFIP1	3.437	8.405	0.028
RBBP8	4.291	5.961	0.002	RPL5P23	1.332	0.263	0.028
TM4SF19	5.398	1.471	0.002	TRAPPC2	15.218	8.3	0.028
SIL1	10.877	15.19	0.002	CD99L2	10.664	11.832	0.028
IGHJ5	100.564	1219.486	0.002	SLC38A10	50.255	59.506	0.028
CYP2D6	2.624	0.096	0.002	ZBTB22	2.975	0.519	0.028
FGFRL1	17.46	4.938	0.002	TVP23C-CDRT4	0.623	0.792	0.028
ZNF600	31.853	8.638	0.002	HCFC1	15.312	16.127	0.028
NEXMIF	0.162	0.058	0.002	ERLIN2	10.541	11.742	0.028
RAB11FIP5	8.603	2.139	0.002	ADRM1	44.526	48.94	0.028
TIMMDC1	21.864	25.814	0.002	ATP23	3.857	6.756	0.028
FP565260.7	11.342	4.246	0.002	COMMD10	17.131	18.354	0.028
ARPC1B	309.005	488.473	0.002	BMP4	0.495	0.016	0.028
FUCA2	14.027	25.334	0.002	GOLGA6L5P	10.044	3.155	0.028
EFTUD2	66.432	77.912	0.002	ATP1B3	64.755	63.753	0.028
ESR2	5.397	1.645	0.002	CDC42SE2	90.404	39.727	0.028
KPTN	4.901	6.276	0.002	ACTB	1985.03	2506.363	0.028
FAM198B	7.296	36.135	0.002	SYCE1L	9.463	2.841	0.028
STUM	3.699	0.082	0.002	CTSH	105.783	196.991	0.028
PLCL1	4.889	1.516	0.002	WNT3	0.287	0.051	0.028
HLA-L	0.027	0.408	0.002	RD3L	0	0.218	0.028
ATP5F1C	78.073	119.313	0.002	CFTR	19.666	6.226	0.028
ALCAM	5.44	13.376	0.002	ZNF573	15.674	6.95	0.028
NOL3	4.122	10.423	0.002	SBK1	12.282	2.961	0.028
AC138969.2	0.005	0.118	0.002	SYNE1	123.097	38.086	0.028
PBXIP1	203.08	67.97	0.002	ACOX2	0.338	0.824	0.028
THOC6	13.85	18.003	0.002	CCDC7	8.588	2.993	0.028
IGHV3-6	0.441	9.442	0.002	RNF216	36.484	15.643	0.028
MRPL28	26.275	34.601	0.002	C4orf50	0.558	0.185	0.028
LRFN2	0.542	0.058	0.002	AIFM1	10.28	12.278	0.028
G6PC3	10.251	14.772	0.002	AC068831.7	2.223	2.651	0.028
BLVRA	27.252	53.242	0.002	SMAD7	36.805	10.546	0.028
NDUFS4	20.209	32.188	0.002	PTBP1	109.567	110.317	0.028
MPP6	4.781	1.744	0.002	ZC3H12B	1.164	0.592	0.028
RNF165	2.997	0.847	0.002	RAB44	3.7	10.636	0.029
TNFRSF17	0.711	10.837	0.002	DDHD2	52.248	25.412	0.029
AC234301.1	1.761	30.044	0.002	SH3RF1	0.701	1.203	0.029
SDC4	1.38	6.75	0.002	AC011295.2	0.811	0.293	0.029
UBE2E1	52.322	65.381	0.002	ERP44	14.043	17.647	0.029
AC245056.4	0.251	4.784	0.002	MDC1	1.369	0.116	0.029
NET1	3.186	9.546	0.002	IGKV1-37	0.415	13.4	0.029
IGKV4-1	23.648	370.069	0.002	TM9SF4	25.294	29.964	0.029
CYFIP1	10.01	25.248	0.002	PPP1CC	85.265	108.017	0.029
WASHC5	20.263	22.179	0.002	CHCHD4	3.138	4.357	0.029
STAC3	8.361	17.863	0.002	ZNF568	9.448	3.873	0.029
PUF60	28.742	31.374	0.002	COPS3	43.003	63.774	0.029
ANG	3.177	12.42	0.002	KBTBD6	1.893	0.77	0.029
HADHB	42.443	70.589	0.002	KPNA5	11.239	4.88	0.029
CBX4	93.65	39.65	0.002	DIAPH3	0.18	0.324	0.029
CAPNS1	274.406	473.047	0.002	GRIP1	4.603	0.643	0.029
CCDC136	10.49	3.742	0.002	FKBP9	8.303	6.973	0.029

Table S1. Continuation

Gene Symbol	Base Mean Control	Base Mean COVID-19 patients	P adj	Gene Symbol	Base Mean Control	Base Mean COVID-19 patients	P adj
MCM10	0.464	1.047	0.002	CCL28	6.963	3.005	0.029
CUEDC2	21.477	26.571	0.002	FAM72C	0.278	0.768	0.029
CCT8	58.648	80.252	0.002	IPO7	18.298	17.122	0.029
AGPAT2	15.66	28.891	0.002	TRGV3	15.144	5.421	0.029
CAPG	52.43	121.274	0.002	PSMB5	10.162	12.166	0.029
FAM72A	2.924	9.064	0.002	ZDHHC11	7.131	2.07	0.029
C14orf119	8.142	11.805	0.002	TRDV2	7.178	1.155	0.029
FKBP5	23.493	110.233	0.002	CASP1	106.02	162.486	0.029
RHOXF1P1	1.037	0.016	0.002	EBI3	0.409	1.871	0.03
C1GALT1C1	4.692	6.2	0.002	NDUFC2	60.745	71.418	0.03
PSMA2	35.753	43.029	0.002	AC079781.1	10.4	3.417	0.03
PPP2R5C	393.623	154.629	0.002	ADGRF1	3.298	0.668	0.03
LILRB5	0.226	1.54	0.002	POLD3	8.412	9.624	0.03
PDSS1	5.552	8.669	0.002	PGGHG	162.242	47.471	0.03
SLC16A1	4.564	5.683	0.002	SP4	7.907	3.215	0.03
ASGR2	11.975	52.723	0.002	PRR5L	122.129	25.39	0.03
GPM6A	1.918	0.204	0.002	P2RX7	8.394	14.07	0.03
SNX3	80.877	124.572	0.002	IFITM4P	0.079	0.946	0.03
LILRB4	0.08	3.073	0.002	HDC	47.382	0.831	0.03
MIB2	121.344	43.502	0.002	SEMA3G	0.281	0.441	0.03
COL6A2	26.606	7.609	0.002	ERH	55.206	53.351	0.03
NAP1L5	6.528	2.574	0.002	ASPH	15.45	36.309	0.03
SH2D1B	17.658	4.591	0.002	LTA	3.634	1.007	0.03
CHCHD1	6.902	11.704	0.002	PGLS	54.387	59.239	0.03
ADAP2	7.502	22.094	0.002	FOXN2	22.553	29.423	0.03
ABCB1	10.015	3.498	0.002	IFI35	32.781	44.361	0.03
RAB11FIP4	22.332	8.673	0.002	NLRP3	17.538	40.69	0.03
NDUFS7	36.318	53.218	0.002	PKD2L2	2.136	0.862	0.03
B4GALNT4	1.807	0.18	0.002	ZNF461	8.518	4.27	0.03
CDK1	1.234	2.73	0.002	PIGS	36.844	36.342	0.031
SAP30	8.292	41.19	0.002	TRAV12-1	26.687	5.523	0.031
MTERF2	10.333	2.763	0.002	APOLD1	4.64	1.31	0.031
PLA2G15	7.516	11.147	0.002	GANC	18.738	28.705	0.031
FGD2	31.437	88.512	0.002	CSTB	22.313	33.68	0.031
OPLAH	1.208	2.758	0.002	AKIP1	4.92	7.659	0.031
LPL	0.518	8.375	0.002	ATP6AP2	105.293	159.579	0.031
COL13A1	1.961	0.125	0.002	PRG4	0.318	0.083	0.031
ZNF330	34.656	52.093	0.002	GMDS	8.911	10.837	0.031
FOPNL	5.693	8.332	0.002	PDHB	18.458	26.708	0.031
MS4A4A	4.532	20.076	0.002	POLDIP3	30.787	33.716	0.031
CLCN7	70.864	101.753	0.002	DOK2	98.828	102.053	0.031
GPNMB	0.784	2.383	0.002	INSR	3.133	7.836	0.031
ANXA7	46.018	56.757	0.002	PAIP1P1	0	0.617	0.031
FAM89A	1.322	3.827	0.003	ITGB2	598.044	750.124	0.031
C3orf22	0.01	0.294	0.003	C14orf132	0.795	0.175	0.031
SMAD1	1.235	5.361	0.003	IL1R2	29.333	497.885	0.031
CD1A	0.441	1.281	0.003	KCNC4	6.535	6.118	0.032
PYCR1	0.539	1.891	0.003	CCR1	7.54	17.197	0.032
FBXO32	23.637	9.514	0.003	NFATC3	51.822	25.63	0.032
ZDHHC20P1	0	1.292	0.003	SLC4A2	38.782	36.209	0.032
IGKV1-6	2.022	16.435	0.003	NSDHL	4.04	4.876	0.032
DHRS4	18.25	25.589	0.003	LRMDA	9.261	11.68	0.032

Table S1. Continuation

Gene Symbol	Base Mean Control	Base Mean COVID-19 patients	P adj	Gene Symbol	Base Mean Control	Base Mean COVID-19 patients	P adj
CCL4L2	1.152	0.056	0.003	ZNF165	0.767	0.252	0.032
MGAT4A	57.135	26.022	0.003	OR1H1P	0.563	0.017	0.032
PLP2	135.102	213.207	0.003	NFATC2	23.121	7.103	0.032
SEC23B	14.488	22.508	0.003	CCL5	474.12	131.801	0.032
TNXB	3.561	2.56	0.003	FGD4	8.67	22.059	0.032
GSC	0.723	0.016	0.003	SLC4A7	30.792	12.001	0.032
NTN4	1.628	0.141	0.003	AC010463.1	0.175	0	0.032
DYNLL1	67.898	112.811	0.003	SPTSSA	5.108	7.701	0.032
TNRC6B	55.748	19.155	0.003	NAP1L3	1.466	0.299	0.032
ADAM15	45.681	94.893	0.003	MX1	34.052	67.566	0.032
ELL2	20.763	36.049	0.003	SPACA6	9.535	3.552	0.032
TBL2	6.737	8.79	0.003	TMEM191A	9.903	3.715	0.032
NTRK1	3.189	0.383	0.003	IL17C	1.619	0.452	0.032
E2F1	1.211	3.305	0.003	SAP25	61.858	28.442	0.032
SEMA6B	0.42	1.593	0.003	RNF39	0	0.312	0.032
IGKV2D-24	0.209	2.868	0.003	RPE	12.168	17.507	0.032
JPH4	0.397	1.21	0.003	HLA-G	0.176	0.005	0.032
TNRC6C	15.345	3.93	0.003	PTDSS1	53.75	61.567	0.032
KRR1	45.348	21.942	0.003	AL353743.1	4.822	2.238	0.032
MGME1	9.01	14.124	0.003	CTIF	2.998	5.256	0.032
PRRT1B	0.975	0.102	0.003	RHOT1	23.433	32.263	0.032
UQCRH	143.823	210.997	0.003	AL357143.1	1.187	0.169	0.032
KIAA0895L	5.799	9.109	0.003	ENO2	28.431	9.791	0.032
DDOST	105.516	104.742	0.003	RORA	103.34	28.618	0.032
PGAM1	85.486	111.352	0.003	CPED1	3.449	8.962	0.032
PNRC1	378.634	162.562	0.003	YWHAE	69.647	115.835	0.032
CXXC4	0.384	0.026	0.003	EXT1	6.076	10.935	0.033
HSD17B4	31.723	58.572	0.003	ATP5MPL	95.029	103.5	0.033
HK1	49.394	81.197	0.003	KCTD14	0.208	0.492	0.033
COQ5	13.952	17.297	0.003	AC104763.3	2.45	0.409	0.033
KLF2	599.034	173.237	0.003	MYL6B	6.949	7.422	0.033
SLC30A4	5.694	2.527	0.003	FAM47E-STBD1	0.256	0.622	0.033
CES1P1	0.004	0.739	0.003	SOCS6	1.966	3.079	0.033
EPHB1	22.314	1.106	0.003	DDX6	83.726	44.194	0.033
VPS35L	19.892	25.82	0.003	STIM2	61.896	29.503	0.033
CALU	17.818	22.969	0.003	TUBGCP5	4.589	1.699	0.033
C3AR1	3.335	10.745	0.003	ADAM1B	0.854	0.186	0.033
HOXB2	13.769	6.465	0.003	PLEKHB2	29.778	34.337	0.033
CRISP3	1.006	0.2	0.003	P2RY1	0.575	1.774	0.033
C1orf162	102.715	224.181	0.003	GPR84	0.347	1.031	0.033
SPON1	2.768	0.903	0.003	C11orf24	9.049	12.509	0.033
MPND	10.694	13.632	0.003	NDUFA6	15.504	27.534	0.033
COMMD9	35.93	63.332	0.003	AC112484.2	1.241	3.948	0.034
SLC45A3	5.67	0.628	0.003	DNAJB1	116.717	40.368	0.034
MATK	77.043	16.698	0.003	KIT	0.698	0.145	0.034
H1F0	9.909	33.939	0.003	AC106865.1	1.214	2.948	0.034
TRGJP1	78.836	7.827	0.003	KRT23	6.696	0.921	0.034
GDI2	99.898	126.733	0.003	ANKRD18A	1.625	1.295	0.034
IGF2BP3	1.743	3.902	0.003	GPN3	4.166	7.042	0.034
NDUFA13	69.488	87.397	0.003	PROCR	5.246	2.261	0.034
TWF2	47.876	56.679	0.003	KIR3DX1	5.446	1.068	0.034

Table S1. Continuation

Gene Symbol	Base Mean Control	Base Mean COVID-19 patients	P adj	Gene Symbol	Base Mean Control	Base Mean COVID-19 patients	P adj
IGKV2D-28	0.28	71.721	0.003	MMP14	2.413	3.201	0.034
MND1	0.293	1.116	0.003	ETS2	25.099	67.033	0.034
HRH4	1.403	0.195	0.003	CCDC88A	28.576	48.18	0.034
ANXA5	101.406	224.399	0.003	GTF2IP23	2.025	6.189	0.034
ALDH3A2	18.55	24.114	0.003	HMGN1P10	1.223	0.102	0.034
KCNK13	0.273	0.979	0.003	JUND	463.916	200.73	0.034
MRPL3	25.466	28.196	0.003	CD38	6.685	25.665	0.034
BEGAIN	2.347	0.807	0.003	CLEC1B	10.306	33.515	0.035
LINC00854	19.652	41.502	0.003	CNNM3	30.756	14.777	0.035
ZDHHC11B	3.165	0.392	0.003	PITPNC1	50.067	20.709	0.035
ECH1	69.761	114.446	0.003	ALDH1L2	0.483	0.598	0.035
ANXA8L1	0.029	0.777	0.003	TNFSF8	16.341	19.892	0.035
HOXB3	10.988	5.203	0.003	FBP1	11.637	16.319	0.035
PLCD1	23.235	8.429	0.003	PTPA	38.384	42.71	0.035
AC078857.1	1.971	0.196	0.003	RIMBP3	0.446	0.08	0.035
LMO2	22.247	54.759	0.003	ERI2	6.005	6.545	0.035
KDELC1	0.134	0.421	0.003	AL031577.1	2.84	0.479	0.035
PTGS2	66.058	4.151	0.003	CEP170	22.719	30.262	0.035
IFI27L2	40.786	60.923	0.003	HK3	57.029	122.801	0.035
HLA-A	283.135	17.087	0.003	KIAA1522	0.89	1.193	0.035
SCIMP	17.151	34.046	0.003	LACC1	1.692	3.15	0.035
LMNB1	17.327	19.316	0.003	FGD6	4.068	5.01	0.035
ARHGEF35	1.747	0.3	0.003	ARMT1	6.028	7.634	0.035
E2F2	2.67	7.784	0.003	SORD	4.763	6.558	0.035
CTSD	234.907	427.984	0.003	TRIM51	0.163	0	0.035
TRIM39	22.876	9.472	0.003	SERPING1	4.003	10.931	0.035
SRP54	26.486	29.87	0.003	FAM151B	11.451	12.453	0.035
HEXB	59.776	104.807	0.004	TULP2	3.619	7.144	0.035
RLN3	0.777	7.669	0.004	GCC2	50.415	21.497	0.035
AC006946.1	12.205	32.087	0.004	SRP9	108.226	136.964	0.036
ARVCF	6.95	2.737	0.004	DGKK	0.587	0.095	0.036
LRFN3	5.81	1.297	0.004	ALAS1	11.302	14.839	0.036
DPYD	33.376	76.908	0.004	PCTP	7.717	10.805	0.036
REELD1	2.119	0.555	0.004	ATP13A1	37.156	38.663	0.036
STS	1.434	5.291	0.004	LAIR1	3.535	18.31	0.036
ZFHX3	65.082	3.863	0.004	EXT2	13.548	13.674	0.036
PDIA2	0.71	0.051	0.004	NPC2	170.525	293.144	0.036
TK2	11.102	17.28	0.004	AGRN	2.9	4.434	0.036
PRR9	0.803	0.007	0.004	EIF4EBP1	13.433	21.584	0.036
VAMP2	168.588	72.954	0.004	JADE3	0.324	0.823	0.036
LCNL1	2.253	0.479	0.004	FTL	2485.724	4783.853	0.036
CASS4	8.154	2.478	0.004	CCDC92	16.127	6.387	0.036
LDLR	31.93	35.572	0.004	GPX1P1	1.577	3.944	0.036
HNRNPAB	47.163	59.19	0.004	EBLN2	4.459	6.332	0.036
KIAA0408	0.684	0.164	0.004	ITGAM	212.245	311.026	0.036
AAR2	9.432	10.412	0.004	VIM	1093.554	1476.11	0.036
CCL4	27.86	3.586	0.004	COX7B	59.263	89.934	0.036
SGCD	0.806	0.105	0.004	RAB34	16.059	23.842	0.036
HIGD2A	122.006	180.4	0.004	BACE1	5.887	8.765	0.036
RPS3AP6	3.531	0.307	0.004	ORAI3	8.247	11.989	0.036
SNX29	11.588	12.193	0.004	CATSPERG	9.068	3.137	0.036
UCHL1	0.026	1.001	0.004	GRN	194.128	419.154	0.036

Table S1. Continuation

Gene Symbol	Base Mean Control	Base Mean COVID-19 patients	P adj	Gene Symbol	Base Mean Control	Base Mean COVID-19 patients	P adj
CPSF3	11.988	16.525	0.004	PRR16	0.219	0.906	0.036
EIF6	34.47	43.237	0.004	HKDC1	1.226	0.287	0.036
AJM1	6.103	1.68	0.004	PRDX4	7.996	14.76	0.036
MAPK6	13.272	16.082	0.004	CPM	9.755	18.16	0.036
RNF17	0.156	0.003	0.004	CLIC1	222.498	334.734	0.036
IGKV5-2	1.348	19.388	0.004	H2AFZP6	3.547	0.79	0.036
IGLC3	42.664	560.804	0.004	GPAT2P1	0.014	0.469	0.036
NME1	8.265	16.526	0.004	TPMT	5.62	6.384	0.036
POU3F3	0.567	0.02	0.004	CMPK2	7.158	17.798	0.036
EPB41L4A	6.388	1.85	0.004	FAR2P1	0.823	0.356	0.036
VARS2	3.407	6.233	0.004	PHF1	107.777	42.509	0.036
ANKRD12	56.695	24.303	0.004	CFAP45	9.474	1.435	0.036
FAM109B	1.946	2.037	0.004	SLC2A4RG	56.601	24.489	0.036
MRPS36	5.263	0.164	0.004	RUFY4	2.896	5.888	0.037
TMCC2	3.19	48.408	0.004	SNX32	1.099	1.741	0.037
LILRA4	2.25	0.147	0.004	VENTX	4.912	12.756	0.037
FEZF2	1.101	0.011	0.004	DNAJC24	15.851	7.707	0.037
TFCP2	15.428	20.222	0.004	TOMM40L	8.369	9.915	0.037
CTSZ	148.109	321.021	0.004	ARSB	6.453	9.835	0.037
HNRNPF	59.524	78.316	0.004	RIOX1	7.216	2.958	0.037
RNASE3	1.582	10.361	0.004	IGHV1-3	13.116	94.901	0.037
GDF11	3.468	0.986	0.004	PPP1R8	10.176	11.864	0.037
STBD1	0.217	0.786	0.004	PAK1IP1	3.854	5.122	0.037
GRHPR	25.922	42.402	0.004	DRAM1	23.835	28.713	0.037
CPE	0.031	0.546	0.004	DYNLT3	15.891	7.753	0.037
AC008758.1	0.453	0.02	0.004	CLCF1	8.125	2.585	0.037
DEFA3	72.895	7.326	0.004	JDP2	18.775	61.934	0.037
CHIC1	5.647	1.813	0.004	TMEM182	29.862	14.691	0.037
STT3A	25.271	37.133	0.004	COA4	20.529	23.081	0.037
JAKMIP2	2.745	0.596	0.004	TXNRD2	17.128	22.922	0.037
KCTD5	10.468	12.493	0.004	RALGDS	84.272	35.884	0.037
AD000671.2	2.583	3.338	0.004	TRBJ1-5	195.696	36.098	0.038
HCCS	6.36	10.399	0.004	MCTS1	22.695	23.847	0.038
WASF1	0.786	2.089	0.004	DNAJB9	32.574	17.99	0.038
ALDOA	384.349	519.075	0.004	IRF8	85.626	172.041	0.038
ALDH9A1	20.265	23.741	0.004	BHLHA15	0.563	3.368	0.038
DARS2	3.83	3.35	0.004	PDK4	3.225	10.412	0.038
IGHJ4	136.47	554.778	0.004	PLIN2	28.284	48.23	0.038
ANGPTL6	4.737	0.939	0.004	PLCB3	5.353	7.23	0.038
SMIM34B	0.423	2.888	0.004	AL355355.2	5.19	1.076	0.038
NRIP1	7.761	13.552	0.004	OAS2	28.177	49.409	0.038
SORT1	10.793	18.17	0.004	FBXO43	0.102	0.261	0.038
C5orf30	1.65	8.15	0.004	TMEM117	2.054	0.584	0.038
AKAP5	1.972	0.591	0.004	BBX	23.837	11.448	0.038
ZHX2	9.92	4.484	0.004	CYB5R1	22.854	31.645	0.038
CLCN4	1.024	1.713	0.004	PMVK	12.62	15.957	0.038
FAM234A	16.167	20.25	0.004	GYG1	26.781	41.827	0.038
TTC4P1	0.939	0.15	0.004	PSMD1	22.323	29.403	0.039
RMI1	1.484	2.583	0.004	ONECUT1	0.459	0.036	0.039
POLE4	37.462	40.708	0.004	SMARCAL1	9.874	10.862	0.039
IGHV3-74	3.903	44.727	0.004	LHFPL5	7.127	2.142	0.039
ITM2A	74.348	18.227	0.004	LBH	68.169	26.193	0.039

Table S1. Continuation

Gene Symbol	Base Mean Control	Base Mean COVID-19 patients	P adj	Gene Symbol	Base Mean Control	Base Mean COVID-19 patients	P adj
SLC1A5	12.187	28.395	0.005	SMCO4	14.397	37.554	0.039
TSHZ2	3.682	0.527	0.005	AC006030.1	0.762	0.293	0.039
TRIM7	2.445	7.65	0.005	EOMES	5.852	2.35	0.039
KIR2DL1	3.442	0.113	0.005	SVIP	19.685	8.049	0.039
ENPP3	1.572	0.195	0.005	ADGRL1	30.153	18.38	0.039
IFI44	57.256	167.689	0.005	RFX8	0.336	0.062	0.039
NBPF13P	0.472	0.048	0.005	MCM4	12.638	17.614	0.039
DESI1	18.577	22.572	0.005	KIR3DL1	0.36	0	0.039
LGALS1	243.178	452.653	0.005	LONRF3	4.543	8.234	0.039
GKAP1	8.246	2.915	0.005	GM2A	21.56	40.733	0.039
CDC42BPB	7.244	10.991	0.005	CCSAP	9.234	4.158	0.04
PKNOX2	0.556	0.014	0.005	NUDT19P5	0.283	0.999	0.04
DERA	13.254	20.339	0.005	PRDM11	3.362	1.697	0.04
FADS1	6.374	10.136	0.005	G6PD	64.627	91.468	0.04
PSMD2	117.591	114.664	0.005	AL031736.2	0.406	0.013	0.04
COX15	13.616	16.129	0.005	FAM156B	2.138	0.539	0.04
UNC13B	0.353	0.763	0.005	HLA-H	0	0.614	0.04
SHMT2	25.552	37.246	0.005	RSF1	46.423	20.474	0.04
HLA-A	7.632	337.878	0.005	AC002996.1	3.97	16.355	0.04
BMPR1A	2.948	1.257	0.005	CCL4L2	4.561	0.472	0.04
PDIA6	43.504	58.92	0.005	MRGBP	4.41	4.958	0.04
WNT5B	0.236	1.088	0.005	SPATA5	3.051	1.451	0.04
DDX41	40.655	50.54	0.005	MEIS2	3.305	0.301	0.04
MT-ND6	3148.22	612.393	0.005	TMEM116	11.79	5.626	0.04
SIGIRR	264.446	80.466	0.005	KLF12	26.32	8.302	0.04
SNX8	8.105	15.579	0.005	FBXL8	9.513	11.154	0.04
IGLJ3	0.678	27.478	0.005	TGFBR1	28.923	14.116	0.04
PTGDS	143.08	34.489	0.005	APCDD1	1.089	1.301	0.04
PER3	5.999	1.831	0.005	QSER1	4.637	5.947	0.04
HCG4B	0.058	0.808	0.005	FBXO6	11.234	14.265	0.04
UTP11	7.458	11.762	0.005	ASB13	3.943	5.574	0.04
C5orf15	22.214	28.556	0.005	AC015818.6	1.017	0.059	0.04
SMARCD3	29.497	57.841	0.005	GINS2	0.79	1.381	0.04
MED8	8.489	11.939	0.005	HIVEP2	16.289	8.9	0.04
C6orf48	26.449	4.088	0.005	SPON2	193.129	65.34	0.04
LPCAT1	79.516	25.72	0.005	AHDC1	11.052	6.015	0.041
PSMB1	74.524	91.178	0.005	EIF4A1	524.811	705.051	0.041
CRIP2	17.778	2.504	0.005	ZNF292	26.416	14.4	0.041
TSHZ1	10.968	3.936	0.005	MRPL47	13.408	16.048	0.041
BTBD9	10.058	4.26	0.005	RACGAP1	3.584	5.696	0.041
LLGL2	57.761	18.119	0.005	SLC9A9	8.362	13.074	0.041
CA11	9.439	3.314	0.005	OR7E13P	0.516	0.071	0.041
SLC25A6	223.089	288.022	0.005	OXTR	1.714	0.641	0.041
CH25H	0.041	0.672	0.005	NFIA	2.231	6.24	0.041
KIF14	0.199	0.404	0.005	CRLF1	7.95	2.827	0.041
TMEM69	9.363	11.656	0.005	TBC1D8	25.396	34.679	0.041
OGFR	41.743	49.532	0.005	RBP7	24.17	80.843	0.041
COL18A1	26.26	5.701	0.005	NCEH1	3.19	4.688	0.041
TMEM121B	0.851	3.115	0.005	EDEM2	19.738	23.104	0.041
GSTM5	0.177	0.007	0.005	EML4	48.739	20.151	0.041
RPL34P20	8.498	2.085	0.005	RHAG	0.658	2.491	0.041
MYH11	11.057	4.075	0.005	AC243837.2	0.479	0.079	0.041

Table S1. Continuation

Gene Symbol	Base Mean Control	Base Mean COVID-19 patients	P adj	Gene Symbol	Base Mean Control	Base Mean COVID-19 patients	P adj
PACS1	103.197	44.99	0.005	GBE1	5.869	8.02	0.041
HGF	2.853	8.417	0.005	GPR153	2.065	0.596	0.041
LRP2BP	1.669	0.647	0.005	CMTM4	2.565	5.493	0.042
FZD2	0.727	2.384	0.006	KCNE3	12.721	28.999	0.042
CRYBG2	5.891	1.809	0.006	TMEM254	9.252	11.113	0.042
TRIM71	0.117	0.583	0.006	PSMC1	58.415	70.294	0.042
KDELR2	28.944	31.701	0.006	XIRP2	0.031	0.372	0.042
IKBKE	18.922	22.025	0.006	EREG	16.773	53.128	0.042
MIOS	14.875	17.84	0.006	PPM1N	5.79	2.212	0.042
RBBP8P1	0.999	0.144	0.006	CXorf57	6.757	2.48	0.042
DRAXIN	1.612	0.579	0.006	SUMO3	47.734	52.876	0.042
BZW2	12.577	18.706	0.006	AC090971.3	4.954	1.407	0.042
SLFN11	24.863	41.85	0.006	STX12	31.088	40.342	0.042
NOL4L	43.293	15.23	0.006	COL8A2	1.104	2.157	0.042
POLR2E	79.737	101.96	0.006	ODF2L	57.547	25.663	0.042
TMEM173	64.587	80.849	0.006	ATP6AP1	51.354	73.64	0.042
CXCL14	2.323	0.028	0.006	ALPK2	0.886	0.094	0.042
UST	3.013	0.867	0.006	CARNS1	12.659	3.63	0.042
CMTM7	30.595	41.204	0.006	MUC16	0.18	0.02	0.043
POP7	9.209	11.503	0.006	ETF1P1	0.239	0	0.043
GSR	23.297	41.3	0.006	TTC16	11.845	2.92	0.043
SDHB	48.225	57.97	0.006	MTND2P40	0.606	0.085	0.043
EVI2A	50.776	62.914	0.006	DBH	1.278	0.272	0.043
NUDT16	12.73	29.268	0.006	UBE2E2	9.867	13.763	0.043
GARS	49.94	48.802	0.006	SPHK1	3.519	9.114	0.043
WDR61	21.817	25.198	0.006	SGSM1	2.049	0.748	0.043
NDUFB3	25.358	29.819	0.006	FNDC11	1.07	0.35	0.043
TDRD9	3.255	10.991	0.006	HLA-DRB6	0.32	1.06	0.043
RPN2	73.483	90.558	0.006	METTL9	5.505	13.425	0.043
TUBGCP2	40.013	42.068	0.006	SNX1	131.746	150.679	0.043
TIMM13	13.822	21.105	0.006	HASPIN	0.188	0.552	0.043
OR2A4	0.241	0	0.006	KBTBD4	17.931	8.41	0.043
SH2D4A	1.148	2.909	0.006	TIMP2	21.547	42.454	0.043
CMAS	12.513	17.472	0.006	GNAL	3.811	1.087	0.043
ZNF91	13.069	5.095	0.006	PDHA1P1	0.984	0.083	0.043
NEBL	2.729	0.693	0.006	MIER2	8.314	11.706	0.043
SDHC	43.285	72.799	0.006	GRIK1	0.347	0.644	0.043
ZNF329	4.447	1.712	0.006	CCT4P2	0.36	0.017	0.043
CCL4	25.815	8.335	0.006	DIAPH2	6.27	8.856	0.043
FAAHP1	10.435	2.428	0.006	MRPL19	9.328	9.837	0.043
ADA2	74.372	123.827	0.006	MPHOSPH8	69.07	27.187	0.044
PPP2R2B	18.129	5.119	0.006	PGM2L1	3.613	0.81	0.044
SOWAHD	0.905	2.014	0.006	ARL6	2.63	1.235	0.044
PYHIN1	54.839	17.235	0.006	EXOSC1	20.63	23.142	0.044
SPTLC2	25.981	43.429	0.006	SAT2	39.386	49.501	0.044
CALCRL	1.882	3.355	0.006	TMX2	28.267	30.47	0.044
MCEMP1	12.24	40.696	0.006	CDT1	1.316	2.149	0.044
ACLY	25.836	29.298	0.006	ASPRV1	2.26	0.532	0.044
TJP3	4.437	1.983	0.006	ANO10	9.899	12.377	0.044
COASY	29.283	33.25	0.006	TMEM258	73.239	98.162	0.044
BFAR	20.812	25.016	0.006	PRSS22	0.507	0.018	0.044
KRT2	0.301	0.04	0.006	AL592183.1	32.828	8.682	0.044

Table S1. Continuation

Gene Symbol	Base Mean Control	Base Mean COVID-19 patients	P adj	Gene Symbol	Base Mean Control	Base Mean COVID-19 patients	P adj
HLA-B	55.045	182.106	0.006	PCLAF	6.251	13.307	0.044
TAS2R20	0	0.219	0.006	DAP	32.338	40.018	0.045
SLITRK4	0.568	2.32	0.006	UGGT2	5.597	5.647	0.045
AP1B1	32.959	47.971	0.007	IGLV3-22	0	2.695	0.045
GART	20.996	20.762	0.007	SUMF1	12.658	15.219	0.045
RPEP4	2.534	0.092	0.007	IQCN	9.807	3.502	0.045
SMKR1	2.454	0.631	0.007	TMEM230	53.009	57.88	0.045
IGKV2-29	2.07	16.288	0.007	RBMS1P1	5.558	18.123	0.045
TRBJ2-3	372.604	66.935	0.007	SELENOM	15.788	4.596	0.045
AC020898.1	2.392	0.145	0.007	AMER2	0.219	0.05	0.045
ARPC1A	25.564	28.205	0.007	ZNF44	25.391	13.29	0.045
CLDN7	2.419	4.183	0.007	AL590240.2	2.065	0.483	0.045
MYO16	0.418	0.086	0.007	BTK	46.436	78.362	0.045
TEX2	6.428	15.075	0.007	MOAP1	44.03	20.156	0.045
RPS6KC1	6.411	8.525	0.007	C1orf226	0.082	0.321	0.045
MARCH1	19.423	53.287	0.007	RCBTB2	25.076	32.275	0.045
PRRT4	0.323	2.845	0.007	FCAR	4.774	5.331	0.045
GHITM	66.651	81.496	0.007	GABRR2	1.635	3.268	0.046
PHEX	0.316	0.064	0.007	ZNF345	12.595	4.864	0.046
TRIM74	2.878	0.93	0.007	CRYBB2P1	18.586	9.727	0.046
MARCH9	23.29	7.948	0.007	GCNT4	1.718	0.496	0.046
IQGAP3	0.067	0.198	0.007	NAGPA	27.509	26.947	0.046
TXN2	31.442	34.894	0.007	PRPF4	7.601	8.165	0.046
C1orf115	0.494	2.04	0.007	NDUFAF4P4	2.668	0.384	0.046
NUP93	29.544	33.083	0.007	SHTN1	8.509	21.841	0.046
NSUN2	46.925	51.014	0.007	SCAI	6.286	2.915	0.046
SEPT10	1.143	3.16	0.007	CS	88.851	84.766	0.046
ADPRH	2.576	6.036	0.007	MRPL53P1	8.15	1.303	0.046
PPEF2	18.137	4.676	0.007	CWF19L2	10.539	3.175	0.046
TRGC1	15.372	3.031	0.007	NAIP	13.423	28.444	0.046
KIF3A	6.031	1.443	0.007	MAP1B	0.224	0.07	0.046
DDB1	55.059	45.626	0.007	IL10RB	32.095	38.547	0.046
KIR2DP1	0.388	0.015	0.007	AOC1	1.353	0.321	0.046
C5	3.338	6.353	0.007	IL4	0.957	0	0.047
KNL1	0.569	1.014	0.007	IFNGR1	51.321	101.731	0.047
SUCLA2	15.245	14.086	0.007	CYBB	98.326	196.249	0.047
TIMP1	70.25	121.722	0.007	SCFD1	42.343	45.772	0.047
WARS2	2.911	3.031	0.007	FAM20A	1.696	2.968	0.047
KIAA1958	1.68	4.79	0.007	AL096701.2	3.503	0	0.047
TMEM150B	12.228	46.292	0.007	GOT2	13.56	15.84	0.047
CORO2B	0.333	0.77	0.007	CPNE2	13.705	23.025	0.047
AC017035.1	1.748	0.212	0.007	PROSER1	28.323	31.65	0.047
FAH	8.808	21.528	0.007	ZNF529	13.691	6.464	0.047
PADI2	7.464	17.468	0.008	SEC24D	12.452	17.167	0.047
IGHV4-55	0.682	9.98	0.008	CD68	130.736	268.543	0.047
KCNN3	0.078	0.487	0.008	AC104763.1	3.636	0.479	0.047
C19orf84	6.059	0.828	0.008	IL17RA	45.103	75.107	0.047
SLC16A8	0.44	3.123	0.008	HMGB3	5.439	9.062	0.047
ANTXRL	5.821	0.25	0.008	PPP1R16B	29.341	10.082	0.048
MPV17L2	3.833	5.425	0.008	ATP6V0D1	65.045	91.574	0.048
NSG1	41.262	27.873	0.008	DPYSL4	0.789	0.201	0.048
MARVELD1	4.32	18.56	0.008	CLIC4	7.713	15.468	0.048

Table S1. Continuation

Gene Symbol	Base Mean Control	Base Mean COVID-19 patients	P adj	Gene Symbol	Base Mean Control	Base Mean COVID-19 patients	P adj
ERO1B	11.726	5.103	0.008	FAM200B	29.246	48.526	0.048
TICRR	0.252	0.49	0.008	TFEC	11.722	34.824	0.048
GCNT1	4.828	11.085	0.008	SMIM4	7.353	14.255	0.048
HNRNPA3P2	2.51	0.235	0.008	DTHD1	12.877	3.439	0.048
ARHGAP31	3.648	8.127	0.008	OPA1	23.513	25.584	0.048
FAM166A	0.263	1.483	0.008	IGLV1-44	11.165	123.721	0.048
PLEKHG3	63.605	16.051	0.008	PTK7	4.433	2.048	0.048
CMTM2	68.733	7.212	0.008	PICK1	17.74	32.778	0.048
PUDP	5.745	10.609	0.008	KY	2.667	0.207	0.048
USP6NL	5.259	7.261	0.008	IGHV3-69-1	0.48	2.642	0.048
ADAM22	2.151	0.385	0.008	SBDS	156.426	72.205	0.048
METTL7A	11.038	38.223	0.008	CAMK1D	20.492	7.93	0.048
OSTC	31.802	47.543	0.008	NUTF2	36.857	41.806	0.048
ALG1	6.367	7.63	0.008	AL163636.2	0.915	5.605	0.048
UQCRC2	108.334	117.058	0.008	CYP19A1	0.011	1.221	0.048
AL356320.2	3.316	0.33	0.008	ATP5MG	150.517	189.556	0.048
TKFC	17.621	27.15	0.008	MED20	5.688	6.447	0.048
MME	39.966	4.179	0.008	ARL8B	30.356	33.002	0.048
IGKV3-7	0.041	3.105	0.008	TUFM	89.008	90.815	0.048
SRGAP2B	5.543	15.488	0.008	AC106047.2	1.471	0.243	0.048
RNF181	42.282	72.321	0.008	B3GLCT	2.108	0.908	0.048
IFNG	25.981	4.965	0.008	MIP	0.284	0.044	0.048
RBM48	12.325	5.399	0.008	ZNF853	1.503	0.481	0.049
F5	8.11	15.101	0.008	PEX10	4.499	4.099	0.049
CCDC80	1.779	0.784	0.008	EMC7	36.99	38.81	0.049
MT1F	6.112	10.604	0.008	TRAPPC11	21.405	24.702	0.049
MCMBP	18.853	24.304	0.008	MITF	7.712	6.499	0.049
LGALS3	73.794	166.636	0.008	CLEC3B	1.993	5.215	0.049
ADORA3	0.282	2.098	0.008	KANSL1	64.89	27.175	0.049
SNX17	56.921	62.939	0.008	EIF2B1	24.814	28.612	0.049
PER1	340.602	520.418	0.008	KL	0.066	0.345	0.049
MYDGF	36.893	58.834	0.008	CSGALNACT2	38.802	65.106	0.049
PSMA5	36.451	43.639	0.008	APP	40.954	59.233	0.049
SOCS7	4.726	4.505	0.008	CFP	104.818	205.473	0.049
EFHC2	2.656	0.78	0.008	MYBPC1	0.055	0.201	0.049
				LRRC37A17P	1.809	0.752	0.049
				CLEC2D	158.124	74.485	0.049
				FAM173B	4.731	5.023	0.049
