# Supplementary material for: Aripiprazole as a Candidate Treatment of COVID-19 Identified Through Genomic Analysis
Source: Front Pharmacol. 2021 Mar 2;12:646701. doi: 10.3389/fphar.2021.646701 (PMC7982825; doi:10.3389/fphar.2021.646701)
Supplement: Supplementary file 2 [file datasheet2.pdf]

Table S2. Differential expression between schizophrenia patiens before and after medication with aripiprazole

Gene Symbol	Base Mean schizophrenia patients (SP) before medication	Base Mean schizophrenia patients after medication	P adj	Gene Symbol	Base Mean schizophrenia patients (SP) after medication	Base Mean schizophrenia atients (SP) after medication	P adj
LMO4	32.086	28.082	0.0039	RAB11FIP3	27.883	31.405	0.0304
ABCA9	0.458	0.420	0.0039	ZCCHC14	29.577	32.640	0.0304
FCN1	1397.628	1161.781	0.0073	MCF2L	8.184	9.280	0.0304
PRRT3	4.883	5.154	0.0094	PCGF1	10.550	10.087	0.0311
HLA-DMA	91.751	82.563	0.0094	ANKRD55	8.395	9.249	0.0312
PRKCQ-AS1	31.743	35.265	0.0094	CEP250	83.042	88.669	0.0312
ZNF764	11.469	12.098	0.0094	DNAJB1	95.632	95.076	0.0320
COMMD3	7.459	7.207	0.0094	MIF	0.328	0.426	0.0320
ARPIN	20.399	19.217	0.0094	WDR41	24.401	22.339	0.0320
STAC3	13.315	10.680	0.0094	ETFDH	15.687	15.005	0.0320
HNMT	22.273	17.993	0.0094	PLEKHA1	50.519	54.384	0.0320
SOCS2	9.135	10.027	0.0094	ZBTB14	33.525	34.835	0.0320
SPOCK1	1.546	1.263	0.0094	EPPK1	3.470	4.345	0.0320
RIN2	24.220	20.029	0.0094	UBQLN4	35.236	36.537	0.0320
CD1D	59.113	53.443	0.0094	RRP8	22.255	22.686	0.0323
TRIM35	27.517	28.405	0.0094	MKRN2OS	0.235	0.328	0.0323
DSC1	3.079	3.172	0.0094	PRRC2B	236.734	261.113	0.0323
PRLR	2.221	2.174	0.0094	TMEM144	7.538	7.473	0.0323
ABCA3	8.610	9.750	0.0094	RHOQ	74.320	69.436	0.0324
ZG16	0.064	0.032	0.0094	HAS3	2.022	2.271	0.0324
DCTN1	108.969	108.604	0.0094	C19orf57	0.673	0.776	0.0325
UQCRHL	4.020	3.572	0.0094	MIF-AS1	0.940	1.047	0.0325
RAC1	154.654	132.773	0.0094	ABCB8	26.044	28.738	0.0325
LILRB4	39.652	31.243	0.0094	MIR4697HG	10.834	13.118	0.0325
FZD2	7.317	6.006	0.0100	FREM3	0.072	0.068	0.0325
HLA-DMB	102.561	96.806	0.0100	NAP1L3	3.828	3.851	0.0325
IBA57	12.737	13.993	0.0100	IL15	11.389	10.781	0.0325
LIMCH1	0.466	0.332	0.0100	TRBJ1-3	2.271	2.901	0.0328
P3H4	2.795	3.052	0.0100	SLA2	44.158	46.380	0.0331
SEC22C	41.216	42.242	0.0100	TRBV6-6	2.812	2.970	0.0331
TRIM65	32.324	33.836	0.0100	NPTN	94.358	87.113	0.0331
GORASP1	42.270	41.405	0.0100	TIMP2	168.603	148.720	0.0331
CNTLN	4.831	4.059	0.0100	MYH9	2548.612	2573.745	0.0331
ATOH8	2.278	1.990	0.0100	VEGFA	16.502	17.879	0.0331
CACNA2D3	7.943	6.666	0.0100	TRBJ1-1	0.563	0.714	0.0337
ADGRD1	7.112	7.220	0.0100	CRHBP	0.807	0.855	0.0342
S100Z	5.794	5.202	0.0100	SLC25A38	34.794	35.604	0.0342
MS4A6A	212.501	180.645	0.0101	UVRAG	41.878	39.284	0.0342
TMEM218	14.404	13.954	0.0101	GPN2	29.167	29.971	0.0346
MORC4	7.822	7.935	0.0107	CTSF	9.285	10.028	0.0347

Table S2. continuation

Gene Symbol	Base Mean SP before med.	Base Mean SP after med.	P adj	Gene Symbol	Base Mean SP before med.	Base Mean SP after med.	P adj
HEXA	15.827	15.709	0.0110	FBXO31	19.502	20.290	0.0347
LY86	37.086	27.581	0.0110	GATA3	34.863	36.700	0.0347
PLEKHO1	167.311	160.997	0.0110	POU6F1	17.843	20.187	0.0347
LRIG1	26.677	29.588	0.0110	GRIN3B	2.572	2.970	0.0351
HFE	11.334	10.160	0.0110	CRELD1	19.873	21.536	0.0352
TMEM106A	11.230	10.499	0.0110	GDF15	0.143	0.142	0.0356
SCNM1	33.147	30.427	0.0110	METTL8	13.655	13.701	0.0357
DCLK2	0.604	0.587	0.0110	THOP1	15.767	16.461	0.0357
PSRC1	6.968	6.826	0.0110	IRAK1BP1	2.512	2.869	0.0357
NAGK	233.370	229.076	0.0110	ZNF662	2.166	2.608	0.0359
PLXNA1	15.086	16.187	0.0110	ATG7	40.809	37.131	0.0360
IL18	8.428	7.262	0.0110	HVCN1	51.901	48.300	0.0361
POM121C	84.068	87.629	0.0111	GINM1	22.296	20.054	0.0368
PCNT	62.176	65.912	0.0119	CATSPER1	4.939	3.989	0.0373
LOXL3	11.364	10.757	0.0120	PPIF	67.630	67.024	0.0373
MUC4	0.400	0.493	0.0121	TICAM2	0.086	0.100	0.0373
RBMS2	18.831	17.822	0.0121	CACNA2D4	15.046	14.328	0.0376
NEGR1	1.656	1.409	0.0123	HEG1	37.917	39.251	0.0376
CXorf21	35.249	30.904	0.0124	ACVRL1	1.796	1.388	0.0376
RASSF4	78.206	74.793	0.0124	MMP28	2.094	2.317	0.0381
DDT	0.357	0.318	0.0124	NAP1L2	5.324	5.331	0.0384
DEFA6	0.041	0.028	0.0124	SYK	315.816	286.552	0.0384
MFSD2A	5.252	5.118	0.0125	UBE2E2	11.989	9.902	0.0384
SMCO4	16.864	13.905	0.0125	LINC01257	0.004	0.002	0.0391
DEFA5	0.035	0.024	0.0132	FOXP3	9.069	10.833	0.0394
BTN2A2	30.211	30.233	0.0140	DECR1	64.052	58.874	0.0397
MMP19	0.960	0.809	0.0140	HHEX	57.354	48.435	0.0397
HS3ST3B1	24.161	25.358	0.0140	SOCS2-AS1	1.080	1.321	0.0397
TMEM150B	10.122	9.003	0.0143	CD33	69.518	64.967	0.0402
PABPC3	0.572	0.556	0.0143	DAPP1	129.732	120.248	0.0402
ZC3H12C	3.894	3.620	0.0144	SLC2A9	9.859	8.894	0.0402
FAM114A1	3.256	2.634	0.0146	SLAIN1	13.737	13.872	0.0403
CD209	1.585	1.465	0.0146	PRRC2A	412.227	423.040	0.0407
ELANE	4.578	5.686	0.0155	ERI2	3.442	3.428	0.0407
DEFA4	8.418	10.398	0.0159	MAP3K9	8.981	10.108	0.0409
CRY1	17.082	17.956	0.0159	AIFM3	5.548	5.118	0.0409
LSS	35.234	39.567	0.0159	ZNF540	7.184	7.704	0.0409
CD1C	16.577	13.710	0.0159	GSN	4.162	3.917	0.0409
KCNK13	2.067	1.680	0.0159	TSHZ2	8.984	9.583	0.0411
C3orf18	8.830	9.927	0.0159	HK3	238.465	220.824	0.0412
VENTX	20.269	19.125	0.0159	NLRC3	114.280	130.972	0.0418
CD5	145.681	158.734	0.0159	FSCN1	6.859	5.858	0.0418
CSTA	52.358	39.948	0.0163	SYT15	0.430	0.427	0.0418

Table S2. continuation

Gene Symbol	Base Mean SP before med.	Base Mean SP after med.	P adj	Gene Symbol	Base Mean SP before med.	Base Mean SP after med.	P adj
DUSP22	92.569	89.484	0.0165	SULT1A2	1.906	1.896	0.0418
SERPINB8	33.849	28.946	0.0165	DMRTC2	0.574	0.537	0.0418
PORCN	11.163	11.876	0.0166	TUB	1.704	2.217	0.0419
ARL10	13.595	15.078	0.0166	ZSWIM5	2.890	3.253	0.0424
ZNF74	11.277	12.353	0.0166	SLC6A19	0.091	0.124	0.0427
SNX30	71.328	68.251	0.0166	ATP6V0E2	23.716	25.220	0.0428
SYNE2	325.571	376.095	0.0166	URGCP	20.728	22.007	0.0428
FXYD3	0.060	0.028	0.0166	CREG1	79.074	66.197	0.0428
SMARCD3	28.463	26.363	0.0166	TBC1D25	28.801	30.253	0.0428
ACOT2	6.989	6.965	0.0166	SIGLECL1	0.089	0.098	0.0428
SOX4	15.660	13.030	0.0166	IGFLR1	4.980	5.291	0.0432
THNSL1	6.760	6.773	0.0166	CD36	228.442	186.978	0.0432
SESN3	151.768	160.200	0.0169	TYMP	256.419	240.394	0.0432
ST6GALNAC6	37.751	41.613	0.0170	DIS3L2	19.860	21.025	0.0435
LMO2	56.784	45.114	0.0175	ZNF503-AS2	0.295	0.240	0.0439
NECTIN4	0.082	0.084	0.0175	ZBTB47	10.560	9.796	0.0439
TRBV24-1	2.195	2.354	0.0176	SMIM10L2A	1.460	1.747	0.0439
SLC27A1	24.356	23.014	0.0181	DIAPH1	502.983	495.157	0.0439
REG3A	0.017	0.009	0.0183	TDP1	25.452	25.620	0.0439
ODF2	34.993	35.675	0.0184	OXNAD1	45.381	51.064	0.0439
HLA-DQB1	155.718	136.808	0.0184	METRN	3.656	3.976	0.0439
HSPB8	0.023	0.009	0.0184	CDX1	0.007	0.006	0.0439
APOA4	0.125	0.028	0.0189	SESTD1	29.783	27.904	0.0440
TMEM30B	3.039	3.405	0.0191	ETHE1	9.315	8.213	0.0441
MPO	15.539	18.985	0.0191	CCDC88C	211.430	228.795	0.0441
FUOM	4.920	4.034	0.0191	CA11	5.406	5.534	0.0450
NCAPD2	89.280	90.487	0.0191	REG1A	0.036	0.023	0.0450
ITPRIPL2	17.633	15.745	0.0191	JOSD1	57.704	59.401	0.0450
KCTD3	6.901	5.865	0.0191	ST3GAL6	24.577	22.171	0.0456
RNF130	237.899	202.855	0.0203	PRDX3	78.875	69.012	0.0456
WAC-AS1	31.920	28.569	0.0203	TMEM205	6.600	5.962	0.0463
PACS1	306.906	319.453	0.0203	SOWAHD	10.636	9.375	0.0463
LPAR1	6.921	6.248	0.0207	C9orf47	1.032	0.931	0.0463
AP1S2	156.667	134.202	0.0207	FUT2	0.669	0.690	0.0463
KLHDC4	58.116	67.823	0.0207	ANOS1	0.230	0.207	0.0463
HGF	10.642	8.899	0.0207	C17orf97	0.737	0.713	0.0463
HAAO	5.326	4.842	0.0209	ITK	219.626	241.973	0.0463
SLC29A2	5.538	6.174	0.0210	POMGNT2	6.469	6.390	0.0463
ANKRD46	13.676	13.275	0.0216	LTA	7.584	7.984	0.0463
KIF5A	0.965	1.155	0.0216	ADAM9	29.477	24.003	0.0463
PREX2	0.040	0.044	0.0216	PCOLCE2	0.256	0.317	0.0463
IDH1	43.647	37.553	0.0216	STAT4	51.194	54.324	0.0463
KCNK6	43.497	42.374	0.0216	TMEM44-AS1	1.701	1.641	0.0463
SCPEP1	129.147	112.695	0.0216	PLA2G4A	6.966	5.735	0.0463
PIGR	0.338	0.216	0.0217	SEC16A	187.700	196.074	0.0463

Table S2. continuation

Gene Symbol	Base Mean SP before med.	Base Mean SP after med.	P adj	Gene Symbol	Base Mean SP before med.	Base Mean SP after med.	P adj
FOLR2	3.965	3.287	0.0217	SLC44A3	0.315	0.289	0.0463
FAM169A	6.976	7.711	0.0219	NLRP3	48.828	42.188	0.0463
MUC2	0.140	0.057	0.0220	INAFM2	12.923	11.596	0.0463
APOB	0.070	0.028	0.0222	LARP7	31.546	29.017	0.0468
TKTL2	0.057	0.059	0.0222	MFSD1	128.547	112.907	0.0468
OSBPL1A	11.526	10.631	0.0222	FABP1	0.093	0.031	0.0472
ITFG1	29.749	26.379	0.0234	CACNA1A	2.349	2.109	0.0472
SLC35B1	22.682	21.435	0.0234	CEP164	45.181	48.135	0.0472
ZNF253	11.584	12.088	0.0234	NDUFA9	6.255	6.223	0.0472
PTPN4	92.680	102.509	0.0234	ALDH1A1	30.859	28.158	0.0473
ADAP2	15.894	13.009	0.0234	PCYT2	15.338	15.874	0.0473
BICDL1	28.958	32.467	0.0234	SELPLG	691.509	663.024	0.0481
ZNF605	14.191	14.984	0.0234	CDC42BPG	8.706	9.682	0.0481
OOSP1	0.090	0.089	0.0234	FGD1	5.298	5.435	0.0481
GALNT11	26.439	27.040	0.0234	TRBV7-2	10.028	10.280	0.0481
TMTC2	12.803	12.145	0.0234	ZFAND5	209.605	187.483	0.0481
GRIK5	0.842	0.961	0.0234	DACH1	4.499	4.619	0.0481
RNASE3	3.636	3.978	0.0234	PRR12	86.912	91.874	0.0481
TAF6	22.898	23.604	0.0235	C1orf162	122.185	107.481	0.0481
FCHSD2	86.882	82.373	0.0235	CCDC102A	7.734	7.803	0.0481
TIGIT	34.527	37.712	0.0236	HPCAL4	8.985	9.611	0.0481
MGST1	13.846	10.642	0.0236	DLG4	18.816	17.104	0.0481
DNAH3	0.352	0.386	0.0236	PHRF1	78.633	82.196	0.0481
TMEM105	0.126	0.116	0.0238	RTN4	202.661	177.860	0.0481
SNX31	0.008	0.007	0.0238	AZU1	5.584	6.858	0.0482
FBXW4	35.133	36.821	0.0238	LTA4H	308.529	268.144	0.0482
CCDC120	8.167	8.962	0.0238	SCIMP	55.445	48.479	0.0482
DGKE	23.170	26.385	0.0238	FGD6	14.422	13.029	0.0482
HEXB	103.561	89.552	0.0238	SLC43A3	16.013	14.203	0.0482
CTSG	2.741	3.349	0.0238	NREP	8.431	7.348	0.0485
EXOC1	60.568	58.185	0.0238	JPH2	0.004	0.006	0.0487
AGFG2	23.771	26.118	0.0238	SEMA5A	1.576	1.520	0.0492
DEFA3	39.095	41.979	0.0238	NFATC2	111.994	122.139	0.0492
KMO	6.715	5.893	0.0238	SEMA4F	7.335	8.218	0.0492
SH2D3A	16.928	18.056	0.0238	AFMID	5.581	5.121	0.0492
ADH1C	0.012	0.005	0.0238	FAM234B	5.543	5.402	0.0492
UGGT2	3.326	2.945	0.0238	GATA6	0.197	0.246	0.0492
ATP8B2	199.796	220.306	0.0238	MYCL	36.146	32.007	0.0492
MS4A4E	4.467	4.517	0.0241	FN3K	1.948	2.045	0.0492
ARHGEF10L	30.471	28.160	0.0241	ZNF276	68.897	76.671	0.0492
P2RY11	4.815	5.502	0.0246	WWC1	0.800	0.817	0.0492
TFEC	43.094	38.676	0.0246	C22orf39	32.830	33.728	0.0492
PDGFRL	0.025	0.023	0.0249	KIAA1210	0.009	0.004	0.0492
ZNF697	5.603	4.916	0.0249	ST3GAL1	172.949	181.783	0.0492
FXYD6	2.689	2.248	0.0249	SPTSSB	2.627	3.174	0.0492

Table S2. continuation

Gene Symbol	Base Mean SP before med.	Base Mean SP after med.	P adj	Gene Symbol	Base Mean SP before med.	Base Mean SP after med.	P adj
MTMR11	25.581	23.881	0.0251	GZMM	39.665	40.225	0.0492
PNMA5	0.466	0.478	0.0252	CLCA1	0.071	0.054	0.0492
ZNF30	4.557	4.711	0.0252	GTF3C1	88.094	91.030	0.0492
CAD	55.617	60.443	0.0252	CMIP	157.473	150.515	0.0492
LRRK1	43.587	43.364	0.0260	KLHL25	4.913	5.518	0.0492
TTC21A	7.321	7.562	0.0271	PLPP1	2.493	2.593	0.0492
TMEM25	7.147	7.883	0.0275	CLDN3	0.023	0.012	0.0492
LRFN3	9.267	9.868	0.0284	SLC24A4	64.710	60.743	0.0492
RUSC2	12.058	10.621	0.0287	EXPH5	2.137	2.437	0.0492
BTK	111.373	100.543	0.0287	FUCA2	16.961	14.433	0.0492
ANKRD63	0.026	0.033	0.0292	C10orf55	0.042	0.037	0.0496
STK38L	80.869	74.721	0.0295	TMTC1	8.613	7.983	0.0497
UBA3	65.395	61.933	0.0295	PTPRE	299.062	290.136	0.0497
MID2	3.703	4.154	0.0298	EMILIN2	157.085	131.873	0.0498
PPP1R13B	13.116	14.169	0.0300	PCED1B	57.908	65.122	0.0499
				SF3B2	247.067	239.019	0.0499
